# Rubisco small subunits from the unicellular green alga *Chlamydomonas* complement Rubisco‐deficient mutants of Arabidopsis

**DOI:** 10.1111/nph.14414

**Published:** 2017-01-13

**Authors:** Nicky Atkinson, Nuno Leitão, Douglas J. Orr, Moritz T. Meyer, Elizabete Carmo‐Silva, Howard Griffiths, Alison M. Smith, Alistair J. McCormick

**Affiliations:** ^1^ SynthSys & Institute of Molecular Plant SciencesSchool of Biological SciencesUniversity of EdinburghEdinburghEH9 3BFUK; ^2^ Department of Metabolic BiologyJohn Innes CentreNorwich Research ParkNorwichNR4 7UHUK; ^3^ Lancaster Environment CentreLancaster UniversityLancasterLA1 4YQUK; ^4^ Department of Plant SciencesUniversity of CambridgeCambridgeCB2 3EAUK

**Keywords:** *Arabidopsis thaliana*, carbon concentrating mechanism (CCM), *Chlamydomonas reinhardtii*, chloroplast, photosynthesis, pyrenoid, Rubisco, tobacco

## Abstract

- Introducing components of algal carbon concentrating mechanisms (CCMs) into higher plant chloroplasts could increase photosynthetic productivity. A key component is the Rubisco‐containing pyrenoid that is needed to minimise CO_2_ retro‐diffusion for CCM operating efficiency.
- Rubisco in Arabidopsis was re‐engineered to incorporate sequence elements that are thought to be essential for recruitment of Rubisco to the pyrenoid, namely the algal Rubisco small subunit (SSU, encoded by *rbcS*) or only the surface‐exposed algal SSU α‐helices.
- Leaves of Arabidopsis *rbcs* mutants expressing ‘pyrenoid‐competent’ chimeric Arabidopsis SSUs containing the SSU α‐helices from *Chlamydomonas reinhardtii* can form hybrid Rubisco complexes with catalytic properties similar to those of native Rubisco, suggesting that the α‐helices are catalytically neutral.
- The growth and photosynthetic performance of complemented Arabidopsis *rbcs* mutants producing near wild‐type levels of the hybrid Rubisco were similar to those of wild‐type controls. Arabidopsis *rbcs* mutants expressing a *Chlamydomonas* SSU differed from wild‐type plants with respect to Rubisco catalysis, photosynthesis and growth. This confirms a role for the SSU in influencing Rubisco catalytic properties.

## Abstract 2

See also the Commentary on this article by Sharwood, **214**: 496–499.

## Introduction

Rubisco (EC 4.1.1.39) catalyses net CO_2_ assimilation in all photosynthetic organisms. Despite this central role, Rubisco is an inefficient enzyme that limits photosynthetic productivity, particularly in plants with the C_3_ photosynthetic pathway. Rubisco has a slow carboxylation rate (*k*_cat_^c^) and a relatively low affinity for CO_2_, with a *K*_m_ for CO_2_ at ambient O_2_ (*K*_c_^air^) close to the CO_2_ concentration in a C_3_ leaf mesophyll cell (Galmés *et al*., 2014). Rubisco also catalyses d‐ribulose‐1,5‐bisphosphate (RuBP) oxygenation, resulting in the energetically expensive photorespiratory pathway where previously fixed CO_2_ is lost (Sharkey, 1988). These features necessitate a large investment in the enzyme (up to 50% of leaf soluble protein) to support adequate rates of CO_2_ assimilation (Parry *et al*., 2013). Increasing the operating efficiency of Rubisco and reducing photorespiration are important approaches for improving yields in C_3_ crop plants (Whitney *et al*., 2011; Parry *et al*., 2013; Carmo‐Silva *et al*., 2015; Long *et al*., 2015; Ort *et al*., 2015).

The operating efficiency of Rubisco in C_3_ plants could be enhanced by elevating the CO_2_ concentration in the chloroplast by means of carbon concentrating mechanisms (CCMs). Possibilities include using components of biochemical CCMs (as in C_4_ and CAM photosynthesis) and/or the biophysical inorganic carbon accumulation mechanisms from cyanobacteria and eukaryotic algae (von Caemmerer *et al*., 2012; Price *et al*., 2013; Meyer *et al*., 2016). In algal CCMs, bicarbonate transporters and localisation of Rubisco and carbonic anhydrase within the chloroplast, and in most instances within the pyrenoid (a microcompartment commonly present in chloroplasts of microalgae), result in saturating CO_2_ concentrations around Rubisco (Morita *et al*., 1998; Giordano *et al*., 2005; Wang *et al*., 2015). Modelling approaches suggest that algal CCMs with a pyrenoid are likely to be more effective in maintaining elevated CO_2_ concentrations around Rubisco than those without (Badger *et al*., 1998). Modelling also reveals that the confinement of Rubisco to a microcompartment would be required for effective operation of a biophysical CCM in a higher plant (Price *et al*., 2013; McGrath & Long, 2014). Recent work has shown that algal CCM components, including carbonic anhydrases and bicarbonate transporters, can be expressed in appropriate subcellular locations in angiosperms (Atkinson *et al*., 2016). To achieve a functional algal CCM in an angiosperm it will also be necessary to introduce a Rubisco capable of assembling into a pyrenoid‐like structure.

Pyrenoid formation in the model green alga *Chlamydomonas reinhardtii* (hereafter Chlamydomonas) depends on the amino acid sequences of the small subunit of Rubisco (SSU, encoded by the *rbcS* nuclear gene family) and, more specifically, on two surface‐exposed α‐helices, which differ markedly between Chlamydomonas and higher plants (Meyer *et al*., 2012). In Chlamydomonas, *rbcS* deletion mutants can be rescued with a SSU variant from angiosperms (Arabidopsis, spinach or sunflower) without compromising *in vitro* Rubisco catalysis (Genkov *et al*., 2010). However, these hybrid Rubisco no longer assembled into a pyrenoid. Accordingly, lines expressing the hybrid Rubisco lacked a functional CCM, resulting in growth only at high CO_2_. Pyrenoid formation and CCM function were restored by expression of a chimeric SSU, where a higher plant SSU was modified with the algal SSU α‐helices (Meyer *et al*., 2012). Thus, assembling a pyrenoid‐like microcompartment in chloroplasts would probably require the incorporation of Chlamydomonas‐like α‐helical sequence into the native angiosperm SSU, in addition to other proteins involved in pyrenoid formation such as the Rubisco‐associated protein EPYC1 (Mackinder *et al*., 2016).

Here we examine how the incorporation of SSUs with α‐helices from Chlamydomonas SSU influences the biogenesis and catalysis of Rubisco in Arabidopsis leaves. The Rubisco large subunits (LSUs, encoded by *rbcL*) harbour the catalytic sites and are highly conserved between algae and angiosperms (Arabidopsis and Chlamydomonas LSUs are 88% identical at the level of amino acid sequences). By contrast, the SSU isoforms of Arabidopsis and Chlamydomonas are only 40–43% identical, even though their tertiary structures are extremely similar, including the positions of the α‐helices (Spreitzer, 2003). Although located on the distal ends of the octameric LSU core of Rubisco and distant from the catalytic sites, the amino acid sequence of the SSUs can affect the catalytic properties of the enzyme (Genkov & Spreitzer, 2009).

In Arabidopsis the SSUs are encoded by four genes. *rbcS1A* on chromosome 1 accounts for ~ 50% of SSU transcript, the remainder being contributed by the *rbcS1B*,* rbcS2B* and *rbcS3B* genes located contiguously on chromosome 5 (Yoon *et al*., 2001). An Arabidopsis double mutant lacking expression of *rbcS1A* and with strongly reduced expression of *rbcS3B* (the *1a3b* mutant) has a low Rubisco content (30% of wild‐type plants) and slow growth (Izumi *et al*., 2012). In this study the *1a3b* mutant was complemented with either the Arabidopsis *rbcS1A* (control), an *rbcS1A* variant encoding the Chlamydomonas α‐helix sequences or the native *rbcS2* gene from Chlamydomonas. We compared the Rubisco content, catalytic properties, leaf photosynthesis and growth of multiple lines for each genotype produced. Our results show that the *1a3b* mutant is a valuable background for attempts to assemble an algal CCM in an angiosperm chloroplast, and for wider examination of the contribution made by SSU genetic diversity to Rubisco properties.

## Materials and Methods

### Plant material and growth conditions

Arabidopsis (*Arabidopsis thaliana* (L.) Heynh. Col‐0) seeds were sown on compost, stratified for 3 d at 4°C and grown at 20°C, ambient CO_2_, 70% relative humidity and 150 μmol photons m^−2 ^s^−1^ in 12 : 12 h light : dark. For comparisons of different genotypes, plants were grown from seeds of the same age and storage history, harvested from plants grown in the same environmental conditions. Tobacco (*Nicotiana benthamiana* L.) was cultivated in a glasshouse (minimum 20°C, natural light supplemented to give light periods of at least 12 h). An Arabidopsis *rbcs1a rbcs2b* mutant (double mutant *1a2b*) was generated by crossing T‐DNA insertion lines GABI_608F01 (At1g67090) and GABI_324A03 (At5g38420). The *1a3b* mutant (GABI_608F01 (At1g67090); SALK_117835 (At5g38410)) was provided by Hiroyuki Ishida, Department of Applied Plant Science, Tohoku University, Japan.

### DNA and RNA extraction, PCR and RT‐qPCR

Genomic DNA was extracted from rosettes according to Li & Chory (1998). PCRs were performed as in McCormick & Kruger (2015) using gene‐specific primers (Supporting Information Table S1). Insertion copy numbers were obtained by quantification of 35S promoter copies (performed by iDNA Genetics, www.idnagenetics.com). mRNA was isolated from the sixth and seventh leaves of 28‐d‐old rosettes and complementary DNA was synthesized with oligo(dT) primers. Reverse transcription quantitative PCR (RT‐qPCR) was carried out as in Andriotis *et al*. (2010). Primers to test for expression of SSU genes were designed to amplify the unique 3′ region of the transcripts (Table S1). Amplification efficiency was determined with a calibration curve for each primer set. Three reference genes (At4g05320 (*UBQ10*), At1g13320 (*PP2A*) and At4g26410 (*RHIP1*) (Czechowski *et al*., 2005)) were used for normalisation. Calculations of relative expression ratios were performed according to Pfaffl (2001).

### Expression of *rbcS* genes in *N. benthamiana* and Arabidopsis *1a3b* mutants

**Figure 1:**
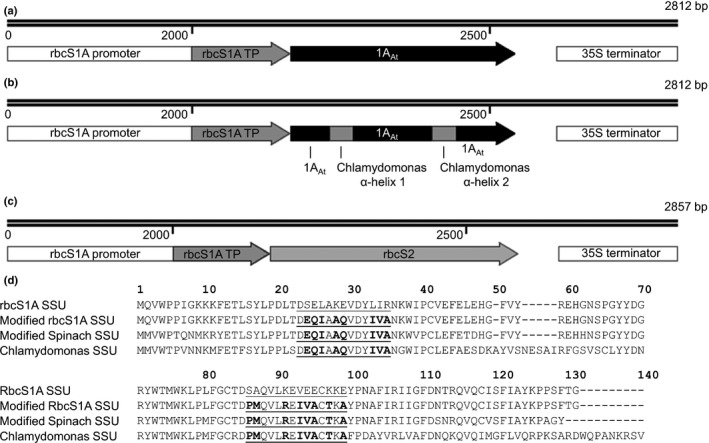
Gene expression cassettes for native and heterologous Rubisco small subunits. *rbcS1A* from *Arabidopsis thaliana* (1A_A__t_) (a), *rbcS1A* with α‐helices from the *Chlamydomonas reinhardtii rbcS* family (1A_A__t_ MOD) (b), and mature *rbcS2* from Chlamydomonas (S2_Cr_) (c) were expressed using the *rbcS1A* promoter (not drawn to scale) and *35S* terminator. For S2_Cr_, the chloroplast transit peptide (TP) of Chlamydomonas rbcS2 (45 amino acids) was replaced with the rbcS1A TP (55 amino acids) from Arabidopsis to facilitate localisation of the mature rbcS2 to the chloroplast. (d) Alignments of the mature SSU peptides generated in this study. Numbering is relative to the Chlamydomonas rbcS2 sequence. Residues that comprise the two α‐helixes A and B are underlined, and those different from rbcS1A are in bold. For comparison with 1A_A__t_ MOD, the modified spinach SSU generated by Meyer *et al*. (2012) is included.

The α‐helices of *rbcS1A* (At1g67090) were replaced with those from the Chlamydomonas *rbcS* family (Fig. 1) using overlapping PCR with Phusion^®^ High‐Fidelity DNA polymerase (as per the manufacturer's instructions; New England BioLabs). The promoter region (2 kb) upstream of *rbcS1A* was fused to the complete cDNA sequences of native or modified SSUs. The *rbcS1A* chloroplast transit peptide (TP) sequence was fused to the mature Chlamydomonas *rbcS2* (Cre02.g120150) (Goldschmidt‐Clermont & Rahire, 1986) cDNA before promoter addition. Promoter–cDNA fusions were cloned into Gateway Entry vectors (pCR^®^8/GW/TOPO^®^TA Cloning^®^ Kit; Thermo Fisher Scientific), then into the binary destination vector pGWB4 (Nakagawa *et al*., 2009) or pB7WG (Karimi *et al*., 2002) (Notes S1). Stop codons were removed to allow in‐frame C‐terminal fusion to a sequence encoding green fluorescent protein (GFP) in pGWB4. Binary vectors were transformed into *Agrobacterium tumefaciens* (AGL1) for transient gene expression in tobacco (Schöb *et al*., 1997) or stable insertion in Arabidopsis plants by floral dipping (Clough & Bent, 1998). Homozygous insertion lines were identified in the T_3_ generation by seedling segregation ratios on Murashige & Skoog (MS) medium (half‐strength) plates containing phosphinothricin (BASTA^®^, final concentration 10 ng μl^−1^) as a selectable marker. Lines used for subsequent analysis were checked for the presence of T‐DNA insertions at the *rbcS1A* and *rbcS3B* loci.

### Protein quantification and Rubisco content

For determination of leaf protein and Rubisco contents on an area basis, soluble protein was extracted from 2 cm^2^ of snap frozen leaf material from 32‐d‐old plants (sixth and seventh leaf) in 500 μl of 50 mM Tricine‐NaOH (pH 8.0), 10 mM EDTA, 1% (w/v) PVP_40_, 20 mM 2‐mercaptoethanol, 1 mM phenylmethylsulfonyl fluoride and 10 μM leupeptin. Following centrifugation at 2380 ***g*** for 5 min at 4°C, soluble protein was quantified using a Bradford‐based assay (Bio‐Rad) against BSA standards (Thermo Fisher Scientific). Rubisco content was determined in an aliquot of the extract via ^14^C‐CABP (carboxy‐d‐arabinitol 1,5‐bisphosphate) binding following incubation with 10 mM NaHCO_3_, 20 mM MgCl_2_ and the addition of 3 μl 12 mM ^14^C‐CABP (37 MBq mmol^−1^) for 25 min at room temperature (Whitney *et al*., 1999).

Subunit ratios were estimated by immunoblotting. Extracts were subjected to sodium dodecyl sulfate–polyacrylamide gel electrophoresis (SDS‐PAGE) on a 4–12% (w/v) polyacrylamide gel (Bolt^®^ Bis‐Tris Plus Gel; Thermo Fisher Scientific), transferred to polyvinylidene fluoride (PVDF) membrane then probed with rabbit serum raised against wheat Rubisco at 1 : 10 000 dilution (Howe *et al*., 1982) followed by Li‐Cor IRDye^®^ 800CW goat anti‐rabbit IgG (Li‐Cor Inc.) at 1 : 10 000 dilution, then viewed on an Li‐Cor Odyssey CLx Imager. The contributions of LSU and SSUs were estimated from a five‐point standard curve of a wild‐type sample of known Rubisco content (0.1–2.4 μg Rubisco).

### Rubisco catalytic properties

Whole 45‐d‐old rosettes (20–30 cm^2^) were rapidly frozen in liquid nitrogen (N_2_) and Rubisco was extracted as described by Prins *et al*. (2016), then activated for 45 min on ice before assays were conducted at 25°C. Catalytic properties of Rubisco from wild‐type and transgenic lines were determined from ^14^CO_2_ consumption, essentially as described by Prins *et al*. (2016) with alterations as per Orr *et al*. (2016), using 40 μl of extract. Six CO_2_ concentrations were used with O_2_ concentrations of 0 and 21%.

Rubisco specificity factor was determined on Rubisco purified from each genotype from *c*. 300 cm^2^ rosette tissue using the method described by Prins *et al*. (2016), with the omission of the final Sephacryl S‐200 step, which was found to be unnecessary for obtaining a clean extract (Orr *et al*., 2016). Rubisco CO_2_/O_2_ specificity (*S*_C/O_) was determined using the method of Parry *et al*. (1989). At least 10 measurements were made on the Rubisco purified from each genotype. Values were normalised based on measurements made in the same experiment on purified wheat (*Triticum aestivum*) Rubisco, which has an established *S*_C/O_ of 100 (Parry *et al*., 1989).

### Chlorophyll quantification

Leaf discs (*c*. 10 mg fresh weight) were frozen in liquid N_2_, powdered, and then mixed with 100 volumes of ice‐cold 80% (v/v) acetone, 10 mM Tris–HCl. Following centrifugation at 17 200 ***g*** for 10 min, chlorophyll was quantified according to Porra *et al*. (1989).

### Measurement of photosynthetic parameters

Gas exchange and chlorophyll fluorescence were determined using a Li‐Cor LI‐6400 portable infra‐red gas analyser with a 6400‐40 leaf chamber on either the sixth or the seventh leaf of 35‐ to 45‐d‐old nonflowering rosettes grown in large pots to generate leaf area sufficient for gas exchange measurements (Flexas *et al*., 2007). For all gas exchange experiments, leaf temperature and chamber relative humidity were 20°C and *c*. 70%, respectively. Gas exchange data were corrected for CO_2_ diffusion from the measuring chamber as in Bellasio *et al*. (2016). Light response curves for net photosynthetic CO_2_ assimilation (*A*) were generated at ambient CO_2_ (400 μmol mol^−1^). A nonrectangular hyperbola was fitted to the light response (Marshall & Biscoe, 1980; Thornley, 1998). The response of *A* to varying sub‐stomatal CO_2_ concentration (*C*_i_) was measured at 1500 μmol photons m^−2 ^s^−1^. To calculate the maximum rate of Rubisco carboxylation (*V*_c,max_) and the maximum photosynthetic electron transport rate (*J*_max_), the *A*/*C*_i_ data were fitted to the C_3_ photosynthesis model as in Ethier & Livingston (2004) using the catalytic parameters *K*_c_^air^ and affinity for O_2_ (*K*_o_) values for wild‐type Arabidopsis Rubisco at 20°C as reported in Walker *et al*. (2013). For estimates of the ratio of Rubisco oxygenase to carboxylase activity (*V*_o_/*V*_c_), leaves were measured under photorespiratory (ambient oxygen (O_2_), 21% (v/v)) or low‐photorespiratory (low O_2_, 2% (v/v)) conditions (Bellasio *et al*., 2014).

Maximum quantum yield of photosystem II (PSII) (*F*_v_/*F*_m_) was measured using a Hansatech Handy PEA continuous excitation chlorophyll fluorimeter (Hansatech Instruments) (Maxwell & Johnson, 2000). Nonphotochemical quenching (NPQ) analyses were performed using a Hansatech FMS1 pulse‐modulated chlorophyll fluorimeter. Rapid light response curves were generated by measuring the fluorescence response to a saturating pulse (applied every 30 s) under increasing levels of actinic light (0–1500 μmol photons m^−2^ s^−1^). Quenching parameters, including NPQ_s_ and NPQ_f_, were derived as in Griffiths & Maxwell (1999).

### Confocal laser scanning microscopy

Leaves were imaged with a Leica TCS SP2 laser scanning confocal microscope (Leica Microsystems) as in Atkinson *et al*. (2016).

## Results

The *1a3b* mutant of Arabidopsis provided a suitable genotype for examining the influence of heterologous SSUs on leaf photosynthesis and growth. Some aspects of the *1a3b* mutant phenotype may reflect loss of distinct Rubisco isoforms (i.e. forms with different SSU compositions), as well as loss of total Rubisco activity. As a first step to evaluate this possibility, a second mutant, lacking expression of *rbcS1A* and a different minor SSU, *rbcS2B* (the *1a2b* mutant) was included in some of the analyses. Quantification of T‐DNA copy numbers indicated that neither double mutant contained T‐DNA insertions other than those at their respective *rbcS* loci.

### Design and targeting of native and heterologous SSUs

Binary vectors were generated to express either the full‐length native Arabidopsis rbcS1A (1A_At_), the mature Chlamydomonas rbcS2 N‐terminally fused to the chloroplast TP sequence from Arabidopsis rbcS1A (S2_Cr_), or the full‐length Arabidopsis rbcS1A modified to contain α‐helices matching the amino acid sequence as those of the Chlamydomonas SSU family (1A_At_MOD) (Fig. 1; Notes S1). Chlamydomonas and Arabidopsis SSU α‐helices have the same number of amino acids, but differ in terms of chemical composition. Expression of the introduced proteins was driven by the promoter of Arabidopsis *rbcS1A*, which has the highest expression level of the Arabidopsis *rbcS* genes (Izumi *et al*., 2012).

To check the subcellular locations of introduced SSUs, they were initially generated as C‐terminal fusions to GFP and transiently expressed in leaves of *N. benthamiana*. Fluorescence microscopy revealed that all three fusion proteins were located in the chloroplast stroma (Fig. S1). Untagged SSUs were then stably expressed in the Arabidopsis *1a3b* mutant.

### Expression levels, leaf protein and Rubisco content of native and heterologous SSU isoforms

**Figure 2:**
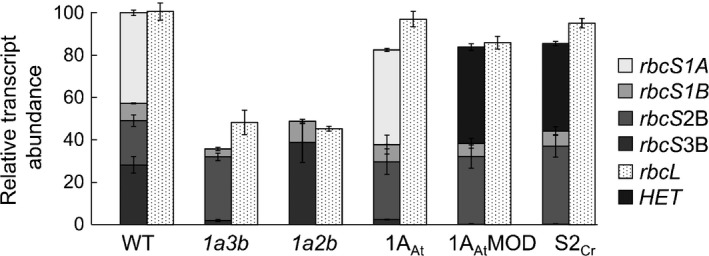
Transcript abundances of the Rubisco gene family in *rbcs* mutants and transgenic lines of *Arabidopsis thaliana*. Abundances of *rbcS1A* (At1g67090), *rbcS1B* (At5g38430), *rbcS2B* (At5g38420), *rbcS3B* (At5g38410) and *rbcL* (Atcg00490) transcripts were quantified relative to wild‐type levels (set at 100) from 28‐d‐old rosettes using RT‐qPCR with gene‐specific primers (Supporting Information Table S1). For wild‐type, *1a3b* and *1a2b* values are the means ± SE of measurements made on three individual 28‐d‐old rosettes. For transgenic lines values are means ± SE of measurements made on nine rosettes, three from each of the three lines. Full expression data are shown in Table S2. *HET*, heterologous *rbcS*.

In wild‐type plants, *rbcS1A* transcripts were the most abundant (43% of the *rbcS* pool), followed by *rbcS3B* (28%), *rbcS2B* (21%) and *rbcS1B* (8%) (Fig. 2; Table S2). The *1a3b* mutant had no detectable transcript for *rbcS1A* and much reduced levels of transcript for *rbcS3B* (*c*. 10% of wild‐type levels). Both *rbcS1A* and *rbcS2B* transcripts were below the level of detection in the *1a2b* mutant. In the *1a3b* mutant, transcript levels for the two undisrupted *rbcS* genes, *rbcS1B* and *rbcS2B*, were 50 and 170% of those in wild‐type plants, respectively. In the *1a2b* mutant, *rbcS1B* and *rbcS3B* transcript levels were 120 and 140% of those in wild‐type plants, respectively. For both mutants, transcript levels for *rbcL* (ATCG00490) and for the overall *rbcS* pool were 50% of those in wild‐type plants.

For each of the three transgenic genotypes expressing native or heterologous SSUs in the *1a3b* mutant background, at least six independent lines segregated in the T_2_ generation. Transgenic plants were screened for faster growth rates and maximum quantum yield of PSII (measured by dark‐adapted leaf fluorescence; *F*_v_/*F*_m_) compared to the *1a3b* mutant (Fig. S2). For further analysis, homozygous T_3_ lines for each genotype were selected from the three best‐performing T_2_ segregating lines.

For each line of each transgenic genotype, transcript levels for the inserted transgene were comparable to those of the native *rbcS1A* gene in wild‐type plants (Fig. 2; Table S2). Levels of transcript of the undisrupted native Rubisco genes were altered in these lines relative to wild‐type plants. For *rbcL*, transcript levels were higher in transgenic than in *1a3b* mutant plants, and in at least one independent line for each construct they were as high as in wild‐type plants. As in *1a3b* mutants, transcript levels for *rbcS2B* in transgenic plants were generally higher than those in wild‐type plants (Fig. 2; Table S2).

**Figure 3:**
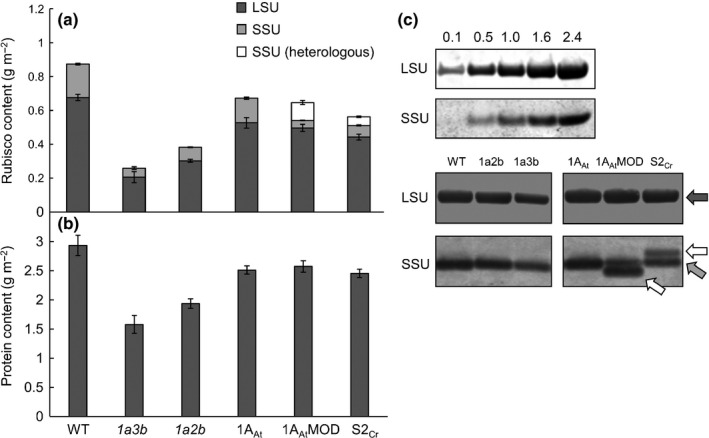
Rubisco and protein contents in *rbcs* mutants and transgenic lines of *Arabidopsis thaliana*. Rubisco (a) and total protein contents (b) are shown for 32‐d‐old plants. Rubisco content was determined via ^14^C‐CABP binding, and subunit ratios were estimated by immunoblotting. For wild‐type, *1a3b* and *1a2b* values are the means ± SE of measurements made on three individual rosettes. For transgenic lines values are means ± SE of measurements made on nine rosettes, three from each of the three lines. (c) Representative immunoblots for wild‐type plants and transgenic lines, probed with a serum containing polyclonal antibodies against Rubisco. Standard curves (0.1–2.4 μg Rubisco) are shown for wild‐type large subunit (LSU, 55 kDa) and small subunits (SSUs, 14.8 kDa), followed by protein amounts in different lines. Native LSU, SSU and heterologous SSUs (15.5 and 14 kDa, respectively) are indicated by dark grey, light grey and white arrows, respectively. Quantification of soluble protein and Rubisco is shown in Supporting Information Table S3.

The leaf Rubisco content in the *1a3b* and *1a2b* mutants was reduced by 70 and 50%, respectively, relative to wild‐type plants (Fig. 3a). Total soluble protein content in leaves of the mutants was also *c*. 60% of wild‐type values in both cases. This reduction was larger than could be accounted for by the reduction in Rubisco content alone (Fig. 3b; Table S3).

Complementation of the *1a3b* mutant restored total Rubisco to 75% of wild‐type levels for 1A_At_ and 1A_At_MOD lines, and to 65% of wild‐type levels for S2_Cr_ lines. Immunoblotting revealed that the heterologous SSUs 1A_At_MOD and S2_Cr_ had different mobilities on SDS‐PAGE gels from the native SSUs (Fig. 3c). This enabled quantification of the relative contributions of the LSU, the native SSUs and the heterologous SSUs to total Rubisco content (Fig. 3a). There were no significant differences in the ratio of LSU to SSU protein between any of the lines tested (Table S3). The 1A_At_MOD and S2_Cr_ transgenic lines retained the same amount of native SSU (i.e. products of the *rbcS1B* and *rbcS2B* genes) as the *1a3b* mutant. Heterologous SSU levels were 2.4‐fold higher than native SSU levels in 1A_At_MOD. By contrast, heterologous SSU levels were 1.4‐fold lower than native SSU levels in S2_Cr_ lines.

### Rubisco activity in mutant and transgenic plants

The *in vitro* catalytic properties of Rubisco from wild‐type plants (Table 1) were in good agreement with those of Galmés *et al*. (2014). The catalytic properties of Rubisco from *1a3b* and *1a2b* mutants were comparable to values for Rubisco from wild‐type plants. Rubisco from 1A_At_ lines had the same catalytic properties as Rubisco from wild‐type plants. This was also true for 1A_At_MOD lines, despite the modification to the Rubisco SSU in these plants. However, *k*_cat_^c^ and *S*_C/O_ values were significantly lower for Rubisco from S2_Cr_ lines than for Rubisco from wild‐type plants.

**Table 1:** Catalytic parameters of Rubisco in *rbcs* mutants and transgenic lines of *Arabidopsis thaliana*

	Wild‐type	*1a3b*	*1a2b*	1A_At_	1A_At_MOD	S2_Cr_
*k*_cat_^c^ (s^−1^)	4.1 ± 0.1	4.2 ± 0.1	4.1 ± 0.2	4.0 ± 0.1	4.1 ± 0.1	3.6 ± 0.1^a^
*K*_c_ (μM)	10.7 ± 0.7	9.5 ± 0.7	9.4 ± 1.1	10.4 ± 1.1	11.5 ± 0.9	9.6 ± 1.0
*K*_c_^air^ (μM)	15.8 ± 1.0	14.3 ± 0.5	15.4 ± 1.5	16.9 ± 1.8	17.1 ± 1.0	16.4 ± 1.2
*k*_cat_^c^/*K*_c_^air^	0.25 ± 0.01	0.3 ± 0.02	0.27 ± 0.02	0.25 ± 0.03	0.24 ± 0.02	0.22 ± 0.03
*S*_C/O_	92.5 ± 1.0 (27)	96.3 ± 1.7 (11)	93.4 ± 1.7 (10)	91.8 ± 1.0 (17)	92.7 ± 0.8 (18)	87.8 ± 0.9^a^ (14)

Rubisco specificity was determined from at least 10 replicate measurements for the enzyme purified from each line. Other catalytic parameters are calculated using the Michaelis–Menten model as described in Prins *et al*. (2016). The table shows mean ± SD values for three biological replicates, except for Rubisco specificity, which is the mean ± SD of the numbers of technical replicates shown in parentheses. All values were measured at 25°C. *K*_c_, *K*_m_ for CO_2_ at 0% O_2_; *K*_c_^air^, *K*_m_ for CO_2_ at 21% O_2_; *k*_cat_^c^, turnover number (mol carboxylation product mol^−1^ active site s^−1^); *k*_cat_^c^/*K*_c_^air^, Rubisco carboxylation efficiency at 21% O_2_; *S*_C/O_, Rubisco specificity factor.

a Significant difference (*P *<* *0.05) as determined by ANOVA followed by Tukey's HSD tests.

### Growth phenotypes

**Figure 4:**
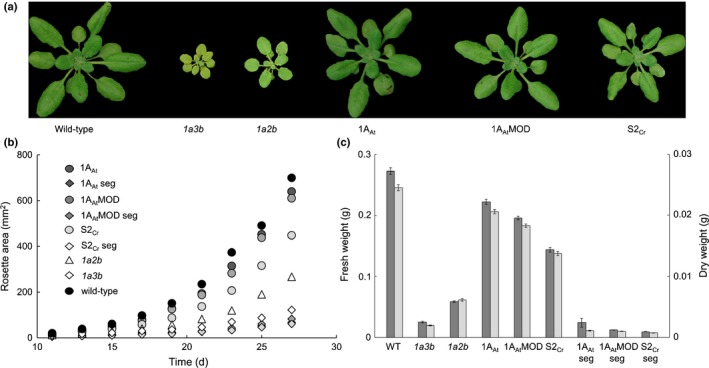
Growth analysis of *rbcs* mutants and transgenic lines of *Arabidopsis thaliana*. (a) Representative examples of 28‐d‐old rosettes (T_3_) for mutants and transgenic genotypes. (b) Rosette expansion of homozygous transgenic and *1a3b* out‐segregant plants compared to that of wild‐type and *1a3b* mutant plants. (c) Fresh and dry weights were compared after 28 d. For wild‐type (WT), *1a3b* and *1a2b* values are the means ± SE of measurements made on 10 individual rosettes. For transgenic lines values are means ± SE of measurements made on 30 rosettes, 10 from each of the three lines. See Supporting Information Table S4 for full dataset. seg, segregating T_3_ wild‐type.

Growth of transgenic lines was compared with that of (1) wild‐type plants, (2) the parental *1a3b* mutant and (3) representative nontransgenic *1a3b* mutant lines selected as out‐segregants from the T_2_ populations (Fig. 4). Fresh and dry weights of the out‐segregant mutant lines were the same as those of the parental *1a3b* mutant at 28 d (Fig. 4c; Table S4). Out‐segregant lines had lower rates of rosette expansion than the parental *1a3b* mutant (Fig. 4b), but this did not affect interpretation of the effects of the transgenes on growth.

As reported previously, *1a3b* mutants had very low growth rates (Izumi *et al*., 2012). All three transgenic genotypes had greater rates of rosette expansion than *1a3b* lines, with 1A_At_ and 1A_At_MOD having higher expansion rates than S2_Cr_ (Fig. 4b). The dry weight of 1A_At_ rosettes at 28 d was on average 84% of that of wild‐type plants, and was not significantly different from the wild‐type for two of the three lines. For 1A_At_MOD and S2_Cr_ lines, dry weight was on average 75 and 56%, respectively, of that of wild‐type plants. There was no significant difference in the ratio of dry weight to fresh weight between wild‐type plants and transgenic lines. All three transgenic genotypes had higher leaf area to weight ratios (rosette area per unit fresh or dry weight) than *1a3b* mutants, and were not significantly different in this respect from wild‐type plants (Table S4).

Rosette expansion rates and fresh and dry weights in the *1a2b* mutant were greater than in the *1a3b* mutant, but lower than those of wild‐type and transgenic lines (Fig. 4c). The *1a2b* mutant had a lower ratio of fresh to dry weight than the *1a3b* mutant (Table S4). Although the specific leaf areas (rosette area per unit dry weight) of *1a2b* and *1a3b* mutants were comparable, rosette area per unit fresh weight was significantly higher in *1a2b* than in *1a3b* mutants.

### Photosynthetic characteristics

**Figure 5:**
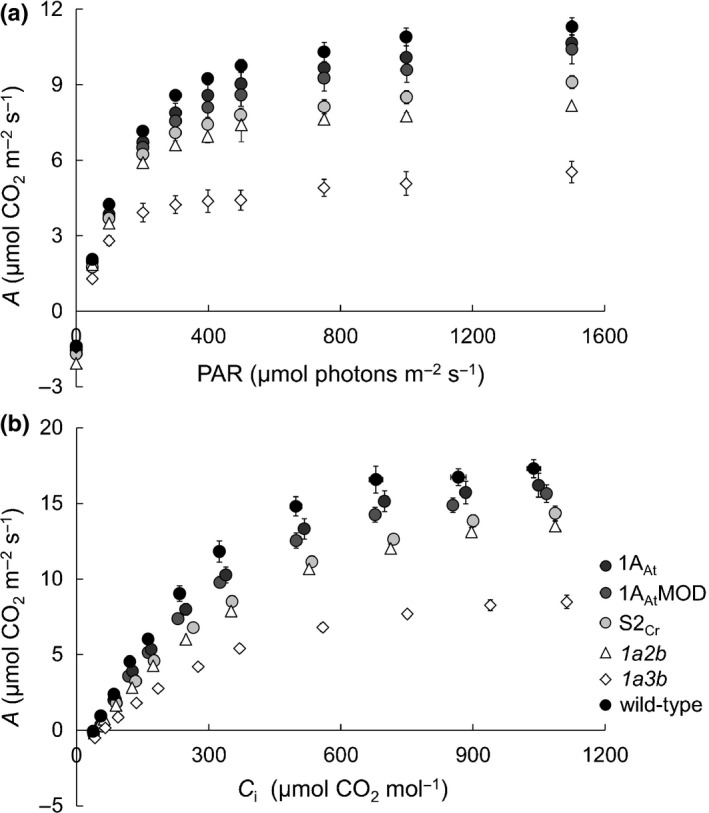
Photosynthesis response curves of *rbcs* mutants and transgenic lines of *Arabidopsis thaliana*. Measurements were made on the sixth or seventh leaf of 35‐ to 45‐d‐old nonflowering rosettes. (a) *A*/PAR curves show the response of CO_2_ assimilation rates to different light levels at ambient CO_2_ levels of 400 μmol mol^−1^. (b) *A*/*C*_i_ curves showing the response of net CO_2_ assimilation to different sub‐stomatal concentrations of CO_2_ (*C*_i_) under saturating light (1500 μmol photons m^−2 ^s^−1^). For wild‐type, *1a3b* and *1a2b* values are the means ± SE of measurements made on individual leaves from four different rosettes. For transgenic lines values are means ± SE of measurements made on 12 rosettes, four from each of the three lines.

At ambient CO_2_ and saturating light, all three transgenic genotypes had much higher rates of CO_2_ assimilation (*A*_max_) than the *1a3b* mutant (*A*/photosynthetically active radiation (PAR) curves, Fig. 5a). *A*_max_ was similar to that of wild‐type plants in 1A_At_ and 1A_At_MOD lines but lower in S2_Cr_ lines (Table 2). *A*_max_ was higher in the *1a2b* than in the *1a3b* mutant, and was comparable in *1a2b* and S2_Cr_ lines. The apparent quantum efficiency (Φ) for all three transgenic lines was higher than in the *1a3b* mutant and comparable with the wild‐type value. Light compensation point and respiration rate in the dark (*R*_d_) were the same in all lines.

**Table 2:** Variables derived from photosynthetic response curves, based on gas exchange analysis of 35‐ to 45‐d‐old *Arabidopsis thaliana* plants

	Wild‐type	*1a3b*	*1a2b*	1A_At_	1A_At_MOD	S2_Cr_
*A*_amb_ (μmol CO_2_ m^−2^ s^−1^)	5.7 ± 0.1^a^	3.4 ± 0.3^c^	4.7 ± 0.1^b^	5.3 ± 0.2^ab^	5.1 ± 0.2^ab^	5.0 ± 0.1^ab^
*A*_max_ (μmol CO_2_ m^−2^ s^−1^)	13.5 ± 0.5^a^	6.8 ± 0.5^c^	10.4 ± 0.2^b^	12.8 ± 0.6^ab^	12.3 ± 0.7^ab^	10.9 ± 0.4^bc^
*g*_s_ (mol CO_2_ m^−2^ s^−1^)	0.34 ± 0.06^a^	0.42 ± 0.06^a^	0.33 ± 0.02^a^	0.3 ± 0.02^a^	0.36 ± 0.03^a^	0.41 ± 0.03^a^
Φ (mmol CO_2_ mol^−1^ photons)	55.9 ± 1.9^a^	42.2 ± 2.3^b^	55.3 ± 1.4^a^	53.5 ± 3.3^a^	51.5 ± 1.8^a^	53.6 ± 0.4^a^
LCP (μmol CO_2_ m^−2^ s^−1^)	16.6 ± 1.3^a^	18.8 ± 0.7^a^	22.7 ± 0.8^a^	18.0 ± 2.6^a^	17.0 ± 1.5^a^	20.9 ± 1.6^a^
*V*_c,max_ (μmol CO_2_ m^−2^ s^−1^)	31.4 ± 1.4^a^	14.6 ± 0.4^d^	20.9 ± 0.4^c^	27.1 ± 1.3^ab^	26.1 ± 1.1^ab^	22.2 ± 0.6^bc^
*J*_max_ (mmol e^− ^m^−2 ^s^−1^)	73.3 ± 2.8^a^	32.8 ± 1.3^d^	53.7 ± 0.9^c^	66.6 ± 3.0^ab^	63.7 ± 2.1^ab^	56.3 ± 1.6^bc^
Γ (μmol CO_2_ mol^−1^)	39.4 ± 2.2^b^	60.0 ± 3.9^a^	42.8 ± 1.6^b^	39.1 ± 0.9^b^	41.4 ± 0.5^b^	43.2 ± 0.8^b^
*R*_d_ (μmol CO_2_ m^−2^ s^−1^)	1.9 ± 0.2^a^	1.8 ± 0.1^a^	2.0 ± 0.1^a^	1.8 ± 0.1^a^	1.9 ± 0.1^a^	1.8 ± 0.1^a^
Initial slope (*A*/*C*_i_)	0.055 ± 0.003^a^	0.024 ± 0.007^d^	0.034 ± 0.006^c^	0.045 ± 0.002^b^	0.044 ± 0.002^b^	0.036 ± 0.001^bc^

For measurements of net photosynthetic CO_2_ assimilation (*A*)/photosynthetically active radiation (PAR), relative humidity was maintained at 68 ± 4% and ambient CO_2_ levels at 400 μmol mol^−1^. For measurements of *A*/sub‐stomatal CO_2_ concentration (*C*_i_), relative humidity was maintained at 73 ± 1% under a constant illumination of 1500 μmol photons m^−2 ^s^−1^. All measurements were performed at 20°C. Values are the mean ± SE of measurements made on four leaves, each from a different plant (as shown in Fig. 5) followed by letters indicating significant differences (*P *<* *0.05) as determined by ANOVA followed by Tukey's HSD tests. Values followed by the same letter are not significantly different. *A*_amb_, net photosynthesis measured at ambient CO_2_ and growth chamber light levels; *A*_max_, light‐saturated CO_2_ assimilation rate at ambient CO_2_; *g*_s_, stomatal conductance to CO_2_ (at ambient CO_2_); Φ, apparent quantum efficiency; LCP, light compensation point; *V*_c,max_, maximum rate of Rubisco carboxylation; *J*_max_, maximum electron transport rate; Γ, CO_2_ compensation point (*C*_i_–*A*); *R*_d_, respiration in the dark.

There were substantial differences between the *1a3b* mutant and the transgenic genotypes in the response of CO_2_ assimilation to changing external CO_2_ concentrations under saturating light (*A*/*C*_i_ curves, Fig. 5b). Several photosynthetic parameters can be derived from *A*/*C*_i_ curves (Table 2). The maximum rate of Rubisco carboxylation (*V*_c,max_) and maximum photosynthetic electron transport rate (*J*_max_) were not significantly different between wild‐type, 1A_At_ and 1A_At_MOD plants, but were lower in S2_Cr_ plants than in wild‐type plants. The initial linear slope of the *A*/*C*_i_ curve (a measure of the carboxylation efficiency and activation state of Rubisco) was lower for transgenic genotypes than for wild‐type plants due to reduced Rubisco content in the transgenic lines (Fig. 3a). In the *1a2b* mutant, *V*_c,max_, *J*_max_, the sub‐stomatal CO_2_ compensation point (Γ) and the initial slope of the *A*/*C*_i_ curve were different from those of the *1a3b* mutant, but similar to values for the S2_Cr_ lines.

Gas exchange rates and chlorophyll fluorescence measurements under photorespiratory (ambient O_2_ (21%)) and nonphotorespiratory (low O_2_ (2%)) conditions were used to derive information about photorespiration (Table 3). Gross CO_2_ assimilation rates (*GA*, CO_2_ assimilation in the absence of respiration) and NADPH production (estimated from the photosynthetic electron transport rate, *J*_NADPH_) can together be used to estimate the ratio of Rubisco oxygenase to carboxylase activity (*V*_o_/*V*_c_) (Bellasio *et al*., 2014).

**Table 3:** Estimates of *in vivo* Rubisco oxygenase and carboxylase activities made from measurements of gas exchange and chlorophyll fluorescence under ambient (21%) or low (2%) O_2_

	Wild‐type	*1a3b*	*1a2b*	1A_At_	1A_At_MOD	S2_Cr_
*GA*_Low_ (μmol m^−2^ s^−1^)	9.48 ± 0.56^a^	4.58 ± 0.4^c^	5.69 ± 0.53^c^	8.73 ± 0.09^ab^	8.57 ± 0.64^ab^	6.72 ± 0.49^bc^
*GA*_amb_ (μmol m^−2^ s^−1^)	6.17 ± 0.36^a^	2.98 ± 0.25^d^	3.67 ± 0.36 ^cd^	5.6 ± 0.15^ab^	5.49 ± 0.42^ab^	4.36 ± 0.38^bc^
*J*_NADPHlow_ (μmol m^−2^ s^−1^)	18.9 ± 1.1^a^	9.1 ± 0.8^c^	11.4 ± 1.1^c^	17.5 ± 0.2^ab^	17.1 ± 1.3^ab^	13.4 ± 0.9^bc^
*J*_NADPHamb_ (μmol m^−2 ^s^−1^)	19.9 ± 0.9^a^	9.2 ± 0.7^c^	11.9 ± 0.9^bc^	18.4 ± 0.2^a^	18.2 ± 1.4^a^	14.1 ± 1^b^
*V*_o_ (μmol m^−2^ s^−1^)	2.21 ± 0.13^a^	1.06 ± 0.11^b^	1.34 ± 0.12^b^	2.09 ± 0.06^a^	2.06 ± 0.17^a^	1.57 ± 0.07^ab^
*V*_c_ (μmol m^−2^ s^−1^)	7.27 ± 0.43^a^	3.52 ± 0.29^d^	4.35 ± 0.41^c^	6.64 ± 0.12^ab^	6.52 ± 0.49^ab^	5.15 ± 0.42^bc^
*V*_o_/*V*_c_	0.304 ± 0.002^a^	0.302 ± 0.013^a^	0.307 ± 0.008^a^	0.313 ± 0.015^a^	0.316 ± 0.012^a^	0.307 ± 0.011^a^

*Arabidopsis thaliana* plants (35–40 d old) were measured under 300 μmol photons m^−2 ^s^−1^, and ambient CO_2_ of 300 μmol mol^−1^ as in Bellasio *et al*. (2014). For wild‐type, *1a3b* and *1a2b* values are the means ± SE of measurements made on individual leaves from five different rosettes. For transgenic lines, values are means ± SE of measurements made on 15 rosettes, five from each of the three lines. Values are followed by letters indicating significant difference (*P *<* *0.05), as determined by ANOVA followed by Tukey's HSD tests. Values followed by the same letter are not significantly different. *GA*_low_, gross photosynthetic rate (*A*+*R*_d_) under 2% O_2_ (2%); *GA*_amb_, gross photosynthetic rate under 21% O_2_; *J*_NADPHlow_, NADPH produced for photosynthesis (derived from electron transport rate) under 2% O_2_; *J*_NADPHamb_, NADPH produced for photosynthesis under 21% O_2_; *V*_o_, Rubisco oxygenation rate; *V*_c_, Rubisco carboxylation rate.

The transgenic genotypes had higher *GA* and *J*_NADPH_ values than the *1a3b* mutant. Values for 1A_At_ and 1A_At_MOD lines were similar to those of wild‐type plants, but values for S2_Cr_ lines were lower. *GA* and *J*_NADPH_ in the *1a2b* mutant were higher than in the *1a3b* mutant, and comparable with values for the S2_Cr_ lines. There were no significant differences in *V*_o_/*V*_c_ values between any of the lines, indicating that relative photorespiratory rates were similar across genotypes under the conditions used.

**Figure 6:**
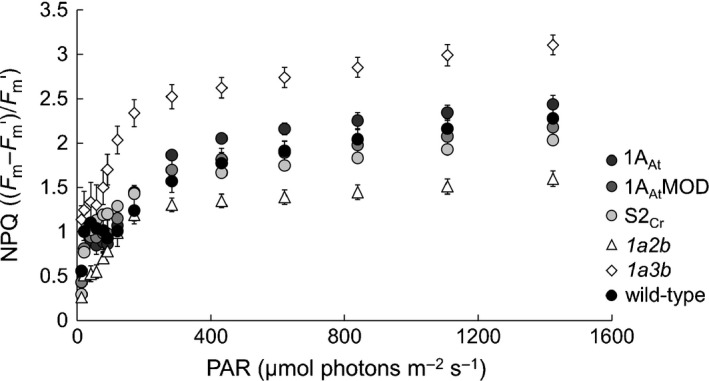
Nonphotochemical quenching response to light in leaves of *rbcs* mutants and transgenic lines of *Arabidopsis thaliana*. All plants were 28 d old. For wild‐type, *1a3b* and *1a2b* values are the means ± SE of measurements made on individual leaves from four different rosettes. For transgenic lines values are means ± SE of measurements on leaves from 12 plants, four from each of the three lines.

Chlorophyll content and dark‐adapted *F*_v_/*F*_m_ values in the transgenic lines and the *1a2b* mutant were higher than in the *1a3b* mutant, and were not significantly different from those of wild‐type plants (Table S5). The *1a3b* mutants had higher levels of NPQ than wild‐type plants, but NPQ in transgenic genotypes was comparable with that of wild‐type plants (Fig. 6). By contrast, the NPQ value for the *1a2b* mutant was lower than that of wild‐type plants. NPQ has two components: fast relaxing quenching (qE: NPQ_fast_) associated with photoprotection, and slow relaxing quenching (qI: NPQ_slow_) associated with chronic photoinhibition (Walters & Horton, 1991). To calculate the contribution of these components in the mutant lines, NPQ was tracked following a period of high light (600 μmol photons m^2^ s^−1^ for 1 h) and subsequent recovery in darkness (1 h). qI was lower in both transgenic genotypes that in wild‐type plants, but qE was elevated in the *1a3b* mutant and reduced in the *1a2b* mutant. The qE : qI ratio was higher in the *1a3b* mutant but lower in the *1a2b* mutant than in wild‐type plants (Table S6).

## Discussion

Our results illustrate the impact of varying Rubisco content and native SSU composition on plant performance in Arabidopsis. Furthermore, we have shown that heterologous, pyrenoid competent SSUs assemble with the native LSU to produce a functional hybrid Rubisco with catalytic properties similar to the native Rubisco. This is a significant step towards the introduction of a functional algal CCM into higher plants.

### Differences in native SSU composition of Rubisco have only minor implications for plant performance in Arabidopsis

The data presented here suggest that the four native SSUs in Arabidopsis are largely equivalent in the properties they convey to the Rubisco enzyme under the growth conditions tested. Four genotypes provided data that lead to this conclusion: (1) wild‐type plants, with the highest Rubisco content and with Rubisco containing almost exclusively rbcS1A, rbcS2B and rbcS3B SSUs (because of its very low transcript levels it is assumed that rbcS1B makes a very minor contribution to the SSU population); (2) 1A_At_ plants, with *c*. 78% of wild‐type Rubisco content and with Rubisco containing mainly rbcS1A and rbcS2B; (3) the *1a2b* mutant, with 45% of wild‐type Rubisco content and with Rubisco containing mainly rbcS3B; and (4) the *1a3b* mutant, with 30% of wild‐type Rubisco content and with Rubisco containing rbcS2B. The catalytic properties *k*_cat_^c^, *K*_c_^air^ and *S*_C/O_ of Rubisco at 25°C were similar in these four genotypes (Table 1), and thus they are largely independent of the native SSU composition of Rubisco in Arabidopsis.

Nearly all the phenotypic differences between the four genotypes with different native SSU compositions can be explained by the differences in total Rubisco content alone. Across these four genotypes, parameters including leaf protein content (Fig. 3; Table S3), the response of photosynthesis to light and to CO_2_ (Fig. 5), Γ and *J*_max_ (Table 2), and the rates of biomass accumulation and rosette expansion (Fig. 4; Table S4) responded to decreasing Rubisco activity in the manner expected for a single enzyme exercising a moderate degree of control over CO_2_ assimilation (Stitt & Schulze, 1994). Additionally, the responses were broadly in line with those observed for tobacco plants with varying amounts of Rubisco activity of probably constant SSU composition (Quick *et al*., 1991; Fichtner *et al*., 1993; Lauerer *et al*., 1993; Stitt & Schulze, 1994), and Arabidopsis plants with strong suppression of expression of all four SSU genes (Zhan *et al*., 2014).

Some features of the four genotypes did not vary consistently with Rubisco content. For example, chlorophyll content, *F*_v_/*F*_m_ and Φ were strongly affected only in the genotype with the lowest levels of Rubisco, *1a3b* (Tables 2, S5). Other parameters including leaf soluble protein content and specific leaf area were affected only in genotypes with less than 50% of wild‐type Rubisco levels (Fig. 3; Table S4). Our data in these respects are reminiscent of those obtained for tobacco under limiting light in which Rubisco activity was varied by expression of antisense RNA that targeted all of the SSUs (Quick *et al*., 1991; Fichtner *et al*., 1993; Lauerer *et al*., 1993; Stitt & Schulze, 1994). Reductions of *c*. 40% or less in Rubisco activity in tobacco plants under limiting light (as in our experiments) had relatively little effect on the rate of photosynthesis and few pleiotropic consequences. Greater reductions progressively affected photosynthesis and downstream processes, different processes being affected at different levels of Rubisco reduction (Quick *et al*., 1991; Stitt & Schulze, 1994). Future experiments will investigate which phenotypic differences between the lines are exaggerated when plants are grown in saturating light.

For processes associated with photoprotection, qualitatively different phenotypes were observed in the *1a3b* and *1a2b* mutants. NPQ was elevated in the *1a3b* mutant. NPQ was also elevated in tobacco and rice with reduced levels of Rubisco (Quick *et al*., 1991; Lauerer *et al*., 1993; Ruuska *et al*., 2000; Ushio *et al*., 2003; von Caemmerer *et al*., 2004): this effect may result from reduced ATP consumption for CO_2_ assimilation, and hence a higher ΔpH across the thylakoid membrane. Lumen acidification promotes activity of the energy‐dissipating xanthophyll cycle (Ruuska *et al*., 2000; Johnson *et al*., 2009; Zaks *et al*., 2012). By contrast, with *1a3b* plants and other plant species with reduced Rubisco, NPQ was reduced in *1a2b* mutants. In particular, *1a2b* plants had a much reduced rate of relaxation of NPQ immediately following the onset of darkness (the qE or fast component of NPQ). The exact mechanism underlying qE is not known (e.g. Johnson *et al*., 2009; Zaks *et al*., 2012). However, as mature rbcS2B and rbcS3B have identical amino acid sequences, the difference in NPQ between the *1a2b* and *1a3b* mutants is likely to stem from the pleiotropic effects of the different degrees of reduction of Rubisco activity in the two mutants, rather than from the different SSU compositions of their Rubiscos.

It is clear from previous work that SSUs can influence Rubisco catalysis. For example, overexpression of specific native or heterologous SSU proteins altered the catalysis of Rubisco in rice leaves, resulting in properties that are more like those of C_4_ plants (i.e. higher *k*_cat_^c^, but also higher *K*_c_ (lower CO_2_ affinity) than for native rice Rubisco) (Ishikawa *et al*., 2011; Morita *et al*., 2014). Over‐expression in Arabidopsis of a pea SSU, differing from Arabidopsis SSUs by 40 amino acids, resulted in Rubisco with slightly reduced carboxylase activity and capacity for activation (Getzoff *et al*., 1998). Similarly, Rubisco properties were changed by introduction of a sorghum SSU into rice (Ishikawa *et al*., 2011). However, except in the case of the rice SSU above, little is known about the functional importance of sequence variation between SSUs within a species. SSU isoforms in a single species are typically very similar. In Chlamydomonas, for example, the two SSUs differ by only four amino acid residues (all outside the α‐helices) and appear to be functionally equivalent (Rodermel *et al*., 1996; Genkov *et al*., 2010). In Arabidopsis, the mature rbcS1A differs from rbcS1B, rbcS2B and rbcS3B by only eight amino acids, six of which are conserved between the three B‐class SSUs (Fig. S3). Two of these are located in the first α‐helix.

### Chlamydomonas‐like SSUs generate a functional hybrid Rubisco in Arabidopsis

Introduction of either a Chlamydomonas SSU (S2_Cr_) or a modified version of rbcS1A (1A_At_MOD) into the Arabidopsis *1a3b* mutant substantially complemented several aspects of the *1a3b* phenotype. In a previous study, a Chlamydomonas SSU introduced into pea chloroplasts was not processed to the mature, active form, probably due to differences in chloroplast import machinery between Chlamydomonas and higher plants (Su & Boschetti, 1994). In this study, replacing the Chlamydomonas SSU TP with the rbcS1A TP directed the mature protein to the chloroplast stroma (Fig. S1). Expression of S2_Cr_ or 1A_At_MOD increased Rubisco content in the *1a3b* mutant without significantly enhancing levels of the remaining native SSUs, and thus both introduced proteins promoted expression of the native LSU and assembled into catalytically active hybrid Rubiscos. These results are consistent with the idea that *rbcL* transcription and LSU synthesis adjust according to the availability of SSU (Wollman *et al*., 1999; Wostrikoff & Stern, 2007; Wostrikoff *et al*., 2012; Zhan *et al*., 2014).

Photosynthesis was restored almost to wild‐type levels in 1A_At_MOD (Fig. 5). Furthermore, the catalytic characteristics of Rubisco in 1A_At_MOD plants, where *c*. 70% of the SSU pool was heterologous, were comparable to those of 1A_At_ and wild‐type plants (Table 1). This suggests that the SSU α‐helix regions alone do not affect Rubisco biogenesis or catalysis, and that Rubisco in higher plants can be made compatible with the requirements of the algal CCM without affecting enzyme performance.

Rubisco in S2_Cr_ plants had lower *k*_cat_^c^ and *S*_C/O_ values than those of wild‐type and 1A_At_MOD Rubisco, even though the S2_Cr_ SSU pool contained only *c*.40% Chlamydomonas SSU. S2_Cr_ lines generally performed less well than 1A_At_MOD lines. Neither S2_Cr_ nor 1A_At_MOD lines are likely to be Rubisco‐limited because they both have *c*. 70% of the Rubisco content of wild‐type plants (Quick *et al*., 1991). Differences in photosynthesis and growth between S2_Cr_ and 1A_At_MOD lines are thus likely to result largely from SSU‐dependent differences in Rubisco catalytic properties. In Chlamydomonas, expression of a higher plant SSU can impart improved catalysis and *S*_C/O_ (Genkov *et al*., 2010). The data shown here demonstrate that the reverse is also true: an algal SSU can negatively affect catalytic properties of the hybrid Rubisco in a higher plant. Since the 1A_At_MOD and S2_Cr_ SSUs have the same α‐helices, differences in catalytic properties of the hybrid enzyme must arise from sequence differences in regions of the SSU outside of these helices.

The Chlamydomonas SSU protein differs in several respects from the Arabidopsis SSUs, including the presence of additional amino acid residues at the C‐terminus and in the loop between β‐strands A and B (Spreitzer, 2003). The latter forms the entrance of the solvent channel and may be important for carboxylation rates and *S*_C/O_ (Karkehabadi *et al*., 1995; Esquivel *et al*., 2013). Hybrid Rubisco enzymes with SSUs that diverge significantly in amino acid sequence from the native SSU frequently have altered stability and properties, and a lower capacity for assembly with the native LSU. The poor complementation of Arabidopsis Rubisco in S2_Cr_ warrants further study to expand upon existing knowledge in this area, including the functional capacity of the chaperone Rubisco activase when presented with hybrid Rubiscos.

### *rbcs* mutants of Arabidopsis are a useful platform for Rubisco analyses and the assembly of an algal CCM

This study shows that Arabidopsis mutants lacking SSU isoforms are a useful platform for attempts to assemble a functional algal CCM in higher plants. Introduction of 1A_At_MOD, containing α‐helices believed to be necessary for pyrenoid assembly, had no apparent effect on Rubisco function and assembly, and plant performance was generally close to wild‐type levels under our growth conditions.

For aggregation of Rubisco into a pyrenoid, additional algal CCM components will be required. Cryo‐electron tomography of Chlamydomonas pyrenoids showed that Rubisco proteins are not randomly arranged, and periodicity is consistent with hexagonal close packing, with a space of 2–4.5 nm between each protein depending on their relative orientations (Engel *et al*., 2015). Other factors, such as linker proteins, are probably needed. Recently, a multiple repeat linker‐protein, EPYC1 (formerly known as LCI5), has been identified in Chlamydomonas that is associated with Rubisco during aggregation within the pyrenoid (Mackinder *et al*., 2016). The 1A_At_MOD and S2_Cr_ Arabidopsis lines are ideal backgrounds in which to test candidates for these other factors as they emerge, to clarify the nature of SSU‐associated interactions, and to integrate other essential algal CCM components (Atkinson *et al*., 2016).

## Author contributions

A.J.M. and N.A. planned and designed the research and wrote the manuscript. A.M.S., D.J.O., M.T.M., H.G. and E.C‐S. assisted in experimental design, data analysis and writing of the manuscript. A.J.M., N.A. and N.L. performed the research, data analysis, collection, and assisted with data interpretation and writing.

## Supporting information

Please note: Wiley Blackwell are not responsible for the content or functionality of any Supporting Information supplied by the authors. Any queries (other than missing material) should be directed to the *New Phytologist* Central Office.

**Fig. S1** Transient expression of Rubisco small subunit–GFP fusion proteins in tobacco.
**Fig. S2** Impact of native and heterologous SSUs on photosynthesis and growth in the Arabidopsis mutant *1a3b* background.
**Fig. S3** Alignments of the mature Arabidopsis SSU amino acid sequences.
**Table S1** Sequences of synthetic oligonucleotides used in this study
**Table S2** Transcript abundances of the Rubisco gene family in *rbcs* mutants and transgenic lines
**Table S3** Rubisco and soluble protein contents for *rbcs* mutants and transgenic lines
**Table S4** Rosette area and biomass for *rbcs* mutants and transgenic lines
**Table S5** Chlorophyll characteristics and maximum quantum yield of PSII (*F*_v_/*F*_m_) for *rbcs* mutants and transgenic lines
**Table S6** Photosynthetic nonphotochemical quenching capacity for *rbcs* mutants

**Notes S1** Expression vectors for Rubisco small subunit (*rbcS*) cassettes.

## References

[nph14414-bib-0001] Andriotis VM , Pike MJ , Bunnewell S , Hills MJ , Smith AM . 2010 The plastidial glucose‐6‐phosphate/phosphate antiporter GPT1 is essential for morphogenesis in Arabidopsis embryos. Plant Journal 64: 128–139. 20659277 10.1111/j.1365-313X.2010.04313.x

[nph14414-bib-0002] Atkinson N , Feike D , Mackinder LC , Meyer MT , Griffiths H , Jonikas MC , Smith AM , McCormick AJ . 2016 Introducing an algal carbon‐concentrating mechanism into higher plants: location and incorporation of key components. Plant Biotechnology Journal 14: 1302–1315. 26538195 10.1111/pbi.12497 PMC5102585

[nph14414-bib-0003] Badger MR , Andrews TJ , Whitney SM , Ludwig M , Yellowlees DC , Leggat W , Price GD . 1998 The diversity and coevolution of Rubisco, plastids, pyrenoids, and chloroplast‐based CO_2_‐concentrating mechanisms in algae. Canadian Journal of Botany 76: 1052–1071.

[nph14414-bib-0004] Bellasio C , Beerling DJ , Griffiths H . 2016 An Excel tool for deriving key photosynthetic parameters from combined gas exchange and chlorophyll fluorescence: theory and practice. Plant, Cell & Environment 39: 1180–1197. 10.1111/pce.12560 25923517

[nph14414-bib-0005] Bellasio C , Burgess SJ , Griffiths H , Hibberd JM . 2014 A high throughput gas exchange screen for determining rates of photorespiration or regulation of C_4_ activity. Journal of Experimental Botany 65: 3769–3779. 25006037 10.1093/jxb/eru238 PMC4085971

[nph14414-bib-0006] von Caemmerer S , Lawson T , Oxborough K , Baker NR , Andrews TJ , Raines CA . 2004 Stomatal conductance does not correlate with photosynthetic capacity in transgenic tobacco with reduced amounts of Rubisco. Journal of Experimental Botany 55: 1157–1166. 15107451 10.1093/jxb/erh128

[nph14414-bib-0007] von Caemmerer S , Quick WP , Furbank RT . 2012 The development of C_4_ rice: current progress and future challenges. Science 336: 1671–1672. 22745421 10.1126/science.1220177

[nph14414-bib-0008] Carmo‐Silva E , Scales JC , Madgwick PJ , Parry MA . 2015 Optimizing Rubisco and its regulation for greater resource use efficiency. Plant, Cell & Environment 38: 1817–1832. 10.1111/pce.12425 25123951

[nph14414-bib-0009] Clough SJ , Bent AF . 1998 Floral dip: a simplified method for *Agrobacterium*‐mediated transformation of *Arabidopsis thaliana* . Plant Journal 16: 735–743. 10069079 10.1046/j.1365-313x.1998.00343.x

[nph14414-bib-0010] Czechowski T , Stitt M , Altmann T , Udvardi MK , Scheible WR . 2005 Genome‐wide identification and testing of superior reference genes for transcript normalization in Arabidopsis. Plant Physiology 139: 5–17. 16166256 10.1104/pp.105.063743 PMC1203353

[nph14414-bib-0011] Engel BD , Schaffer M , Kuhn Cuellar L , Villa E , Plitzko JM , Baumeister W . 2015 Native architecture of the Chlamydomonas chloroplast revealed by *in situ* cryo‐electron tomography. eLife 4: e04889. 10.7554/eLife.04889 PMC4292175 25584625

[nph14414-bib-0012] Esquivel MG , Genkov T , Nogueira AS , Salvucci ME , Spreitzer RJ . 2013 Substitutions at the opening of the Rubisco central solvent channel affect holoenzyme stability and CO_2_/O_2_ specificity but not activation by Rubisco activase. Photosynthesis Research 118: 209–218. 24014091 10.1007/s11120-013-9916-0

[nph14414-bib-0013] Ethier GJ , Livingston NJ . 2004 On the need to incorporate sensitivity to CO_2_ transfer conductance into the Farquhar‐von Caemmerer‐Berry leaf photosynthesis model. Plant, Cell & Environment 27: 137–153.

[nph14414-bib-0014] Fichtner K , Quick WP , Schulze ED , Mooney HA , Rodermel SR , Bogorad L , Stitt M . 1993 Decreased ribulose‐1,5‐bisphosphate carboxylase‐oxygenase in transgenic tobacco transformed with ‘antisense’ rbcS. V. Relationship between photosynthetic rate, storage strategy, biomass allocation and vegetative plant growth at three different nitrogen supplies. Planta 190: 332–345.

[nph14414-bib-0015] Flexas J , Ortuno MF , Ribas‐Carbo M , Diaz‐Espejo A , Florez‐Sarasa ID , Medrano H . 2007 Mesophyll conductance to CO_2_ in *Arabidopsis thaliana* . New Phytologist 175: 501–511. 17635225 10.1111/j.1469-8137.2007.02111.x

[nph14414-bib-0016] Galmés J , Kapralov MV , Andralojc PJ , Conesa MÀ , Keys AJ , Parry MAJ , Flexas J . 2014 Expanding knowledge of the Rubisco kinetics variability in plant species: environmental and evolutionary trends. Plant, Cell & Environment 37: 1989–2001. 10.1111/pce.12335 24689692

[nph14414-bib-0017] Genkov T , Meyer M , Griffiths H , Spreitzer RJ . 2010 Functional hybrid Rubisco enzymes with plant small subunits and algal large subunits: engineered rbcS cDNA for expression in Chlamydomonas. Journal of Biological Chemistry 285: 19833–19841. 20424165 10.1074/jbc.M110.124230 PMC2888394

[nph14414-bib-0018] Genkov T , Spreitzer RJ . 2009 Highly conserved small subunit residues influence Rubisco large subunit catalysis. Journal of Biological Chemistry 284: 30105–30112. 19734149 10.1074/jbc.M109.044081 PMC2781565

[nph14414-bib-0019] Getzoff TP , Zhu GH , Bohnert HJ , Jensen RG . 1998 Chimeric *Arabidopsis thaliana* ribulose‐1,5‐bisphosphate carboxylase/oxygenase containing a pea small subunit protein is compromised in carbamylation. Plant Physiology 116: 695–702. 9489016 10.1104/pp.116.2.695 PMC35128

[nph14414-bib-0020] Giordano M , Beardall J , Raven JA . 2005 CO_2_ concentrating mechanisms in algae: mechanisms, environmental modulation, and evolution. Annual Review of Plant Biology 56: 99–131. 10.1146/annurev.arplant.56.032604.144052 15862091

[nph14414-bib-0021] Goldschmidt‐Clermont M , Rahire M . 1986 Sequence, evolution and differential expression of the two genes encoding variant small subunits of ribulose bisphosphate carboxylase/oxygenase in *Chlamydomonas reinhardtii* . Journal of Molecular Biology 191: 421–432. 3820291 10.1016/0022-2836(86)90137-3

[nph14414-bib-0022] Griffiths H , Maxwell K . 1999 In memory of C. S. Pittendrigh: does exposure in forest canopies relate to photoprotective strategies in epiphytic bromeliads? Functional Ecology 13: 15–23.

[nph14414-bib-0023] Howe CJ , Auffret AD , Doherty A , Bowman CM , Dyer TA , Gray JC . 1982 Location and nucleotide sequence of the gene for the proton‐translocating subunit of wheat chloroplast ATP synthase. Proceedings of the National Academy of Sciences, USA 79: 6903–6907. 10.1073/pnas.79.22.6903 PMC347242 16593250

[nph14414-bib-0024] Ishikawa C , Hatanaka T , Misoo S , Miyake C , Fukayama H . 2011 Functional incorporation of sorghum small subunit increases the catalytic turnover rate of Rubisco in transgenic rice. Plant Physiology 156: 1603–1611. 21562335 10.1104/pp.111.177030 PMC3135941

[nph14414-bib-0025] Izumi M , Tsunoda H , Suzuki Y , Makino A , Ishida H . 2012 RBCS1A and RBCS3B, two major members within the Arabidopsis RBCS multigene family, function to yield sufficient Rubisco content for leaf photosynthetic capacity. Journal of Experimetnal Botany 63: 2159–2170. 10.1093/jxb/err434 PMC3295403 22223809

[nph14414-bib-0026] Johnson MP , Perez‐Bueno ML , Zia A , Horton P , Ruban AV . 2009 The zeaxanthin‐independent and zeaxanthin‐dependent qE components of nonphotochemical quenching involve common conformational changes within the photosystem II antenna in Arabidopsis. Plant Physiology 149: 1061–1075. 19011000 10.1104/pp.108.129957 PMC2633848

[nph14414-bib-0027] Karimi M , Inze D , Depicker A . 2002 GATEWAY vectors for *Agrobacterium*‐mediated plant transformation. Trends in Plant Science 7: 193–195. 11992820 10.1016/s1360-1385(02)02251-3

[nph14414-bib-0028] Karkehabadi S , Peddi SR , Anwaruzzaman M , Taylor TC , Cederlund A , Genkov T , Andersson I , Spreitzer RJ . 1995 Chimeric small subunits influence catalysis without causing global conformational changes in the crystal structure of ribulose‐1,5‐bisphosphate carboxylase/oxygenase. Biochemistry 44: 9851–9861. 10.1021/bi050537v 16026157

[nph14414-bib-0029] Lauerer M , Saftic D , Quick WP , Labate C , Fichtner K , Schulze ED , Rodermel SR , Bogorad L , Stitt M . 1993 Decreased ribulose‐1,5‐bisphosphate carboxylase/oxygenase in transgenic tobacco transformed with ‘antisense’ *rbcS*. VI. Effect on photosynthesis in plants grown at different irradiance. Planta 190: 332–345.

[nph14414-bib-0030] Li J , Chory J . 1998 Preparation of DNA from Arabidopsis. Methods in Molecular Biology 82: 55–60. 9664412 10.1385/0-89603-391-0:55

[nph14414-bib-0031] Long SP , Marshall‐Colon A , Zhu XG . 2015 Meeting the global food demand of the future by engineering crop photosynthesis and yield potential. Cell 161: 56–66. 25815985 10.1016/j.cell.2015.03.019

[nph14414-bib-0032] Mackinder LCM , Meyer MT , Mettler‐Altmann T , Chen VK , Mitchell MC , Caspari O , Rosenzweig ESF , Pallesen L , Reeves G , Itakura A *et al* 2016 A repeat protein links Rubisco to form the eukaryotic carbon‐concentrating organelle. Proceedings of the National Academy of Sciences, USA 113: 5958–5963. 10.1073/pnas.1522866113 PMC4889370 27166422

[nph14414-bib-0033] Marshall B , Biscoe PV . 1980 A model for C_3_ leaves describing the dependence of net photosynthesis on irradiance. Journal of Experimental Botany 31: 29–39.

[nph14414-bib-0034] Maxwell K , Johnson GN . 2000 Chlorophyll fluorescence – a practical guide. Journal of Experimental Botany 51: 659–668. 10938857 10.1093/jxb/51.345.659

[nph14414-bib-0035] McCormick AJ , Kruger NJ . 2015 Lack of fructose 2,6‐bisphosphate compromises photosynthesis and growth in Arabidopsis in fluctuating environments. Plant Journal 81: 670–683. 25602028 10.1111/tpj.12765

[nph14414-bib-0036] McGrath JM , Long SP . 2014 Can the cyanobacterial carbon‐concentrating mechanism increase photosynthesis in crop species? A theoretical analysis. Plant Physiology 164: 2247–2261. 24550242 10.1104/pp.113.232611 PMC3982776

[nph14414-bib-0037] Meyer MT , Genkov T , Skepper JN , Jouhet J , Mitchell MC , Spreitzer RJ , Griffiths H . 2012 Rubisco small‐subunit α‐helices control pyrenoid formation in Chlamydomonas. Proceedings of the National Academy of Sciences, USA 109: 19474–19479. 10.1073/pnas.1210993109 PMC3511088 23112177

[nph14414-bib-0038] Meyer MT , McCormick AJ , Griffiths H . 2016 Will an algal CO_2_‐concentrating mechanism work in higher plants? Current Opinion in Plant Biology 31: 181–188. 27194106 10.1016/j.pbi.2016.04.009

[nph14414-bib-0039] Morita E , Abe T , Tsuzuki M , Fujiwara S , Sato N , Hirata A , Sonoike K , Nozaki H . 1998 Presence of the CO_2_‐concentrating mechanism in some species of the pyrenoid‐less free‐living algal genus *Chloromonas* (Volvocales, Chlorophyta). Planta 204: 269–276. 9530871 10.1007/s004250050256

[nph14414-bib-0040] Morita K , Hatanaka T , Misoo S , Fukayama H . 2014 Unusual small subunit that is not expressed in photosynthetic cells alters the catalytic properties of Rubisco in rice. Plant Physiology 164: 69–79. 24254313 10.1104/pp.113.228015 PMC3875826

[nph14414-bib-0041] Nakagawa T , Ishiguro S , Kimura T . 2009 Gateway vectors for plant transformation. Plant Biotechnology 26: 275–284.

[nph14414-bib-0042] Orr DJ , Alcântara A , Kapralov MV , Andralojc PJ , Carmo‐Silva E , Parry MAJ . 2016 Surveying Rubisco diversity and temperature response to improve crop photosynthetic efficiency. Plant Physiology 172: 707–717. 27342312 10.1104/pp.16.00750 PMC5047088

[nph14414-bib-0043] Ort DR , Merchant SS , Alric J , Barkan A , Blankenship RE , Bock R , Croce R , Hanson MR , Hibberd JM , Long SP *et al* 2015 Redesigning photosynthesis to sustainably meet global food and bioenergy demand. Proceedings of the National Academy of Sciences, USA 112: 8529–8536. 10.1073/pnas.1424031112 PMC4507207 26124102

[nph14414-bib-0044] Parry MA , Andralojc PJ , Scales JC , Salvucci ME , Carmo‐Silva AE , Alonso H , Whitney SM . 2013 Rubisco activity and regulation as targets for crop improvement. Journal of Experimental Botany 64: 717–730. 23162118 10.1093/jxb/ers336

[nph14414-bib-0045] Parry MAJ , Keys AJ , Gutteridge S . 1989 Variation in the specificity factor of C_3_ higher plant Rubiscos determined by the total consumption of Ribulose‐P2. Journal of Experimental Botany 40: 317–320.

[nph14414-bib-0046] Pfaffl MW . 2001 A new mathematical model for relative quantification in real‐time RT‐PCR. Nucleic Acids Research 29: e45. 11328886 10.1093/nar/29.9.e45 PMC55695

[nph14414-bib-0047] Porra RJ , Thompson WA , Kriedemann PE . 1989 Determination of accurate extinction coefficients and simultaneous equations for assaying chlorophylls a and b extracted with four different solvents: verification of the concentration of chlorophyll standards by atomic absorption spectroscopy. Biochimica et Biophysica Acta (BBA) – Bioenergetics 975: 384–394.

[nph14414-bib-0048] Price GD , Pengelly JJ , Forster B , Du J , Whitney SM , von Caemmerer S , Badger MR , Howitt SM , Evans JR . 2013 The cyanobacterial CCM as a source of genes for improving photosynthetic CO_2_ fixation in crop species. Journal of Experimental Botany 64: 753–768. 23028015 10.1093/jxb/ers257

[nph14414-bib-0049] Prins A , Orr DJ , Andralojc PJ , Reynolds MP , Carmo‐Silva E , Parry MAJ . 2016 Rubisco catalytic properties of wild and domesticated relatives provide scope for improving wheat photosynthesis. Journal of Experimental Botany 67: 1827–1838. 26798025 10.1093/jxb/erv574 PMC4783365

[nph14414-bib-0050] Quick WP , Schurr U , Fichtner K , Schulze ED , Rodermel SR , Bogorad L , Stitt M . 1991 The impact of decreased Rubisco on photosynthesis, growth, allocation and storage in tobacco plants which have been transformed with antisense rbcS. Plant Journal 1: 51–58.

[nph14414-bib-0051] Rodermel S , Haley J , Jiang CZ , Tsai CH , Bogorad L . 1996 A mechanism for intergenomic integration: abundance of ribulose bisphosphate carboxylase small‐subunit protein influences the translation of the large‐subunit mRNA. Proceedings of the National Academy of Sciences, USA 93: 3881–3885. 10.1073/pnas.93.9.3881 PMC39453 8632983

[nph14414-bib-0052] Ruuska SA , von Caemmerer S , Badger MR , Andrews TJ , Price GD , Robinson SA . 2000 Xanthophyll cycle, light energy dissipation and electron transport in transgenic tobacco with reduced carbon assimilation capacity. Australian Journal of Plant Physiology 27: 289–300.

[nph14414-bib-0053] Schöb H , Kunz C , Meins F Jr . 1997 Silencing of transgenes introduced into leaves by agroinfiltration: a simple, rapid method for investigating sequence requirements for gene silencing. Molecular and General Genetics 256: 581–585. 9413443 10.1007/s004380050604

[nph14414-bib-0054] Sharkey TD . 1988 Estimating the rate of photorespiration in leaves. Physiologia Plantarum 73: 147–152.

[nph14414-bib-0055] Spreitzer RJ . 2003 Role of the small subunit in ribulose‐1,5‐bisphosphate carboxylase/oxygenase. Archives of Biochemistry and Biophysics 414: 141–149. 12781765 10.1016/s0003-9861(03)00171-1

[nph14414-bib-0056] Stitt M , Schulze D . 1994 Does Rubisco control the rate of photosynthesis and plant growth? An exercise in molecular ecophysiology. Plant, Cell & Environment 17: 465–487.

[nph14414-bib-0057] Su Q , Boschetti A . 1994 Substrate‐ and species‐specific processing enzymes for chloroplast precursor proteins. Biochemical Journal 300: 787–792. 8010961 10.1042/bj3000787 PMC1138235

[nph14414-bib-0058] Thornley J . 1998 Dynamic model of leaf photosynthesis with acclimation to light and nitrogen. Annals of Botany 81: 421–430.

[nph14414-bib-0059] Ushio A , Makino A , Yokota S , Hirotsu N , Mae T . 2003 Xanthophyll cycle pigments and water‐water cycle in transgenic rice with decreased amounts of ribulose‐1,5‐bisphosphate carboxylase and the wild‐type rice grown under different N levels. Soil Science and Plant Nutrition 49: 77–83.

[nph14414-bib-0060] Walker B , Ariza LS , Kaines S , Badger MR , Cousins AB . 2013 Temperature response of *in vivo* Rubisco kinetics and mesophyll conductance in *Arabidopsis thaliana*: comparisons to *Nicotiana tabacum* . Plant, Cell & Environment 36: 2108–2119. 10.1111/pce.12166 23869820

[nph14414-bib-0061] Walters RG , Horton P . 1991 Resolution of components of non‐photochemical chlorophyll fluorescence quenching in barley leaves. Photosynthesis Research 27: 121–133. 24414575 10.1007/BF00033251

[nph14414-bib-0062] Wang Y , Stessman DJ , Spalding MH . 2015 The CO_2_ concentrating mechanism and photosynthetic carbon assimilation in limiting CO_2_: how Chlamydomonas works against the gradient. Plant Journal 82: 429–448. 25765072 10.1111/tpj.12829

[nph14414-bib-0063] Whitney SM , Houtz RL , Alonso H . 2011 Advancing our understanding and capacity to engineer nature's CO_2_‐sequestering enzyme, Rubisco. Plant Physiology 155: 27–35. 20974895 10.1104/pp.110.164814 PMC3075749

[nph14414-bib-0064] Whitney SM , von Caemmerer S , Hudson GS , Andrews TJ . 1999 Directed mutation of the Rubisco large subunit of tobacco influences photorespiration and growth. Plant Physiology 121: 579–588. 10517850 10.1104/pp.121.2.579 PMC59421

[nph14414-bib-0065] Wollman F‐A , Minai L , Nechushtai R . 1999 The biogenesis and assembly of photosynthetic proteins in thylakoid membranes. Biochimica et Biophysica Acta (BBA) – Bioenergetics 1411: 21–85. 10216153 10.1016/s0005-2728(99)00043-2

[nph14414-bib-0066] Wostrikoff K , Clark A , Sato S , Clemente T , Stern D . 2012 Ectopic expression of Rubisco subunits in maize mesophyll cells does not overcome barriers to cell type‐specific accumulation. Plant Physiology 160: 419–432. 22744982 10.1104/pp.112.195677 PMC3440216

[nph14414-bib-0067] Wostrikoff K , Stern D . 2007 Rubisco large‐subunit translation is autoregulated in response to its assembly state in tobacco chloroplasts. Proceedings of the National Academy of Sciences, USA 104: 6466–6471. 10.1073/pnas.0610586104 PMC1851044 17404229

[nph14414-bib-0068] Yoon M , Putterill JJ , Ross GS , Laing WA . 2001 Determination of the relative expression levels of Rubisco small subunit genes in Arabidopsis by rapid amplification of cDNA ends. Analytical Biochemistry 291: 237–244. 11401297 10.1006/abio.2001.5042

[nph14414-bib-0069] Zaks J , Amarnath K , Kramer DM , Niyogi KK , Fleming GR . 2012 A kinetic model of rapidly reversible nonphotochemical quenching. Proceedings of the National Academy of Sciences, USA 109: 15757–15762. 10.1073/pnas.1211017109 PMC3465407 22891305

[nph14414-bib-0070] Zhan GM , Li RJ , Hu ZY , Liu J , Deng LB , Lu SY , Hua W . 2014 Cosuppression of RBCS3B in Arabidopsis leads to severe photoinhibition caused by ROS accumulation. Plant Cell Reports 33: 1091–1108. 24682522 10.1007/s00299-014-1597-4

