# Supplementary material for: Rubisco small subunits from the unicellular green alga Chlamydomonas complement Rubisco‐deficient mutants of Arabidopsis
Source: New Phytol. 2017 Jan 13;214(2):655–67. doi: 10.1111/nph.14414 (PMC5363358; doi:10.1111/nph.14414)
Supplement: Supplementary file 1 — Fig. S1 Transient expression of Rubisco small subunit–GFP fusion proteins in tobacco. Fig. S2 Impact of native and heterologous SSUs on photosynthesis and growth in the Arabidopsis mutant 1a3b background. Fig. S3 Alignments of the mature Arabidopsis SSU amino acid sequences. Table S1 Sequences of synthetic oligonucleotides used in this study Table S2 Transcript abundances of the Rubisco gene family in rbcs mutants and transgenic lines Table S3 Rubisco and soluble protein contents for rbcs mutants and transgenic lines Table S4 Rosette area and biomass for rbcs mutants and transgenic lines Table S5 Chlorophyll characteristics and maximum quantum yield of PSII (F v/F m) for rbcs mutants and transgenic lines Table S6 Photosynthetic nonphotochemical quenching capacity for rbcs mutants [file NPH-214-655-s001.pdf]

New Phytologist

New Phytologist Supporting Information

Article title: Rubisco small subunits from the unicellular green alga *Chlamydomonas* complement Rubisco-deficient mutants of Arabidopsis.

Authors: Nicky Atkinson, Nuno Leitão, Douglas J. Orr, Moritz T. Meyer, Elizabete Carmo-Silva, Howard Griffiths, Alison M. Smith and Alistair J. McCormick

Article acceptance date: 24 November 2016

The following Supporting Information is available for this article:

Notes S1 Expression vectors for Rubisco small subunit (*rbcS*) cassettes (see separate Notes S1.zip file). Gateway destination vector pB7WG (Karimi *et al*., 2002) was used for stable Agrobacterium-mediated insertion into Arabidopsis. For fluorescent tag-based localisation in tobacco, *rbcS* genes were fused to a sequence encoding a GFP tag using destination vector pGWB4 (Nakagawa *et al*., 2009), to produce C-terminally GFP-tagged fusion protein.

Fig. S1 Transient expression of Rubisco small subunit-GFP fusion proteins in tobacco.

Fig. S2 Impact of native and heterologous SSUs on photosynthesis and growth in the Arabidopsis mutant *1a3b* background.

Fig. S3 Alignments of the mature Arabidopsis SSU amino acid sequences.

Table S1 Sequences of synthetic oligonucleotides used in this study

Table S2 Transcript abundances of the Rubisco gene family in *rbcs* mutants and transgenic lines

Table S3 Rubisco and soluble protein contents for *rbcs* mutants and transgenic lines

Table S4 Rosette area and biomass for *rbcs* mutants and transgenic lines

Table S5 Chlorophyll characteristics and maximum quantum yield of PSII (F_v/F_m) for *rbcs* mutants and transgenic lines

Table S6 Photosynthetic nonphotochemical quenching capacity for *rbcs* mutants

Fig. S1 Transient expression of Rubisco small subunit-GFP fusion proteins in tobacco. Tobacco (*Nicotiana benthamiana* L.) was cultivated in a glasshouse (minimum 20°C, natural light supplemented to give light periods of at least 12 h). Plants were *c*. 21-d-old at the time of infiltration, and leaves were imaged between 2 and 7 d after infiltration. Native ($1A_{At}$) and heterologous ($1A_{At}MOD$, $S2_{Cr}$) SSUs are shown. Magenta and green signals are chlorophyll autofluorescence and GFP fluorescence respectively. Overlaid images of these signals are shown: overlaps are white. Bar, 25 µm.

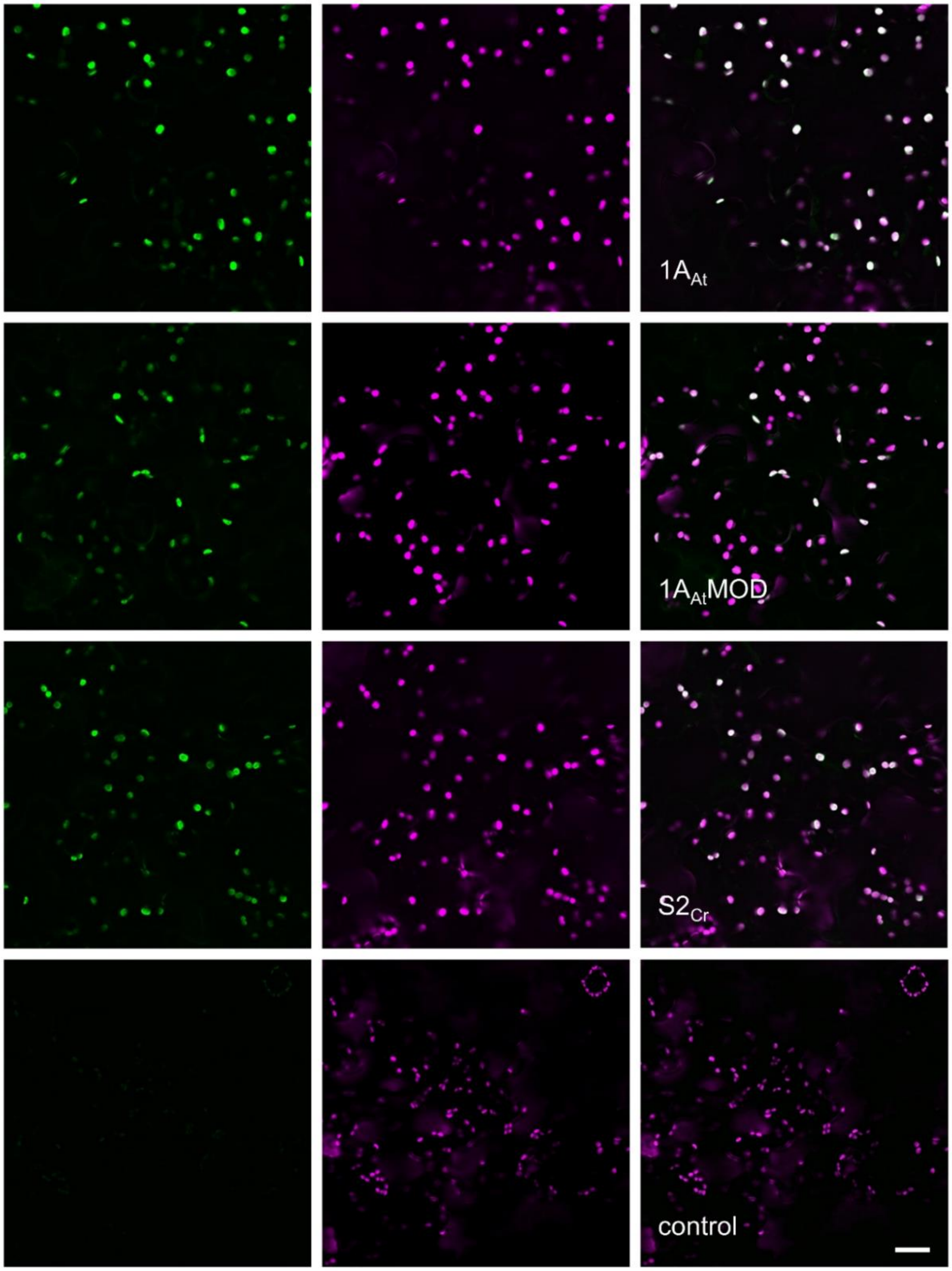

Fig. S2 Impact of native and heterologous SSUs on photosynthesis and growth in the *Arabidopsis thaliana* mutant *1a3b* background. Transgenic lines were screened in the T_2 segregating generation (1:2:1) for differences in growth and maximum quantum yield of PSII (F_v/F_m) relative to *1a3b* mutants and wild-type plants. Values are shown for 45 individual rosettes of wild-type and *1a3b* genotypes, and 15 individual rosettes from each of 6 different transgenic lines (i.e. 90 plants) for $1A_{At}$, $1A_{At}MOD$ and $S2_{Cr}$ genotypes.

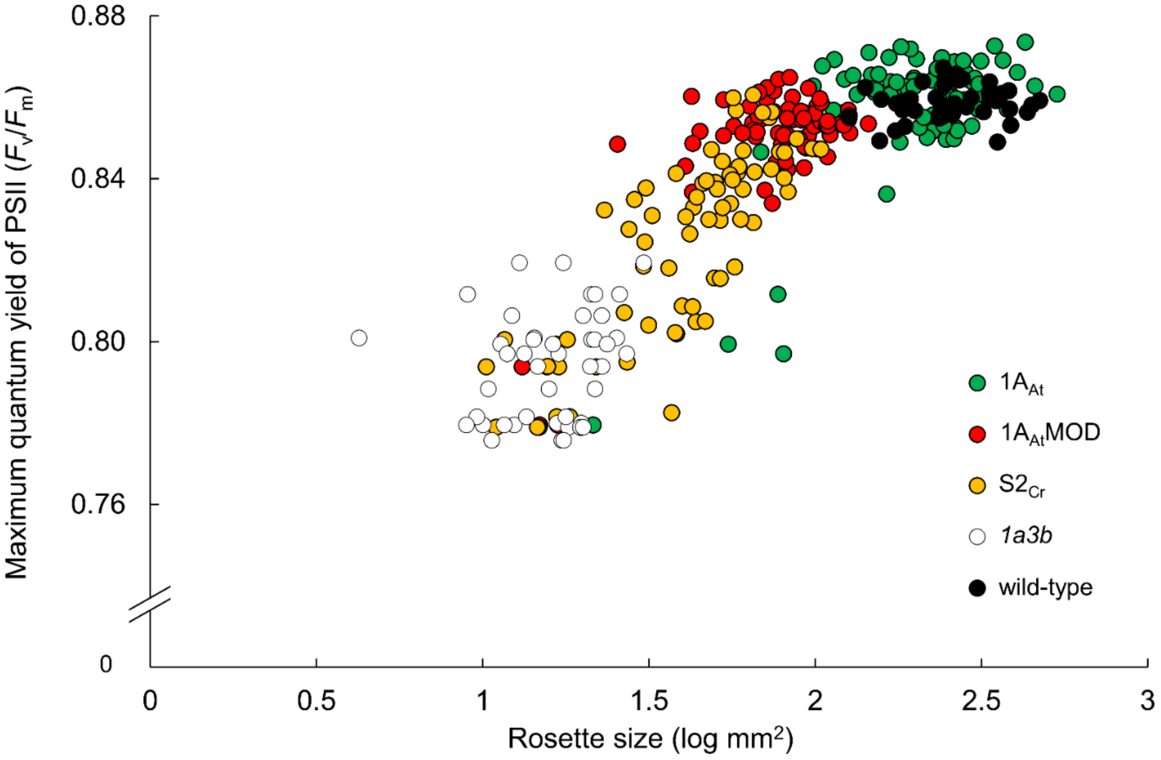

Fig. S3 Alignment of the mature *Arabidopsis thaliana* SSU amino acid sequences. The two α-helixes A and B are highlighted in grey. Differences in amino acid residues between SSUs are in bold and underlined for rbcS1A (1A). Absence of residues is indicated with a dash, and non-conservative differences are marked with a star. Peptides for rbcS1A, rbcS1B (1B), rbcS2B (2B) and rbcS3B (3B) correspond to At1g67090.1, At5g38430.1 At5g38420.1 and At5g38410.1, respectively.

```
1A      MQVWPPIGKKKFETLSYLPDLTDSELAKEVDYLIRNKWIPCVEFELEHGFVYREHGNSPG
1B      MKVWPPIGKKKFETLSYLPDLTDVELAKEVDYLLRNKWIPCVEFELEHGFVYREHGNTPG
2B      MKVWPPIGKKKFETLSYLPDLSDVELAKEVDYLLRNKWIPCVEFELEHGFVYREHGNTPG
3B      MKVWPPIGKKKFETLSYLPDLSDVELAKEVDYLLRNKWIPCVEFELEHGFVYREHGNTPG
                               *

1A      YYDGRYWTMWKLPLFGCTDSAQVLKEVEECKKEYPNAFIRIIGFDNTRQVQCISFIAYKP
1B      YYDGRYWTMWKLPLFGCTDSAQVLKEVEECKKEYPGAFIRIIGFDNTRQVQCISFIAYKP
2B      YYDGRYWTMWKLPLFGCTDSAQVLKEVEECKKEYPGAFIRIIGFDNTRQVQCISFIAYKP
3B      YYDGRYWTMWKLPLFGCTDSAQVLKEVEECKKEYPGAFIRIIGFDNTRQVQCISFIAYKP
                                           *

1A      PSFTG-
1B      PSFTDA
2B      PSFTEA
3B      PSFTEA
            **
```

Table S1 Sequences of synthetic oligonucleotides used in this study.

Primer ID	Forward primer	Reverse Primer	Amplicon	Reference
rbcS1A (GABI_608F01)	CCATAAGGAAAGGGCCAAGT	CATTGTCCAGTACCGTCCATC	980 bp	signal.salk.edu/tdnaprimers.2.html
rbcS2B (GABI_324A03)	TGGGTTCCTCTTGTTCATCAG	CACTTGTTGCGGAGAAGGTAG	1066 bp	signal.salk.edu/tdnaprimers.2.html
rbcS3B (SALK_117835)	TTTTTAAGAGCATCTCGAATCTATCTC	CACTTGTTGCGGAGAAGGTAG	1160 bp	signal.salk.edu/tdnaprimers.2.html
SALK T-DNA (left border)	ATTTTGCCGATTTCGGAAC	-	-	signal.salk.edu/tdna_FAQs.html
GABI T-DNA (left border)	ATATTGACCATCATACTCATTGC	-	-	www.gabi-kat.de/duplofaq/confirmation-strategy.html
rbcS1A (RT-qPCR)	AATTTCCGGACTTAACGTTTGTTT	CATCAGACAGTTGAGAATCCGATAGA	69 bp	(Izumi *et al.*, 2012)
rbcS1B (RT-qPCR)	GCCAAAGTGAAAAAACTGAAGGTT	AAGAGCAGAAATGAAGTGATATGAATAGA	83 bp	(Izumi *et al.*, 2012)
rbcS2B (RT-qPCR)	ACCCATTTCTATGTGGTCAATGC	TTCACTTTCAAACAATAGTTCCTCAAC	80 bp	(Izumi *et al.*, 2012)
rbcS3B (RT-qPCR)	CCTATTGTCTGTGTTCTTTTTCTCTTTATG	TCAAGACGCACGGATATATAAATTACA	99 bp	(Izumi *et al.*, 2012)
rbcL (RT-qPCR)	GATGGGCTTACCAGCCTTGA	CTGGAACGGGCTCGATGT	61 bp	(Izumi *et al.*, 2012)
$1A_{At}$ and $1A_{At}$MOD (RT-qPCR)	TCATTGCCTACAAGCCACCA	CCGCGGGATATCACCACTTT	85 bp	This work
$S2_{Cr}$ (RT-qPCR)	GTGCAGATCATGGGCTTCCT	TACACGGAGCGCTTGTTGG	77 bp	This work
PP2A (RT-qPCR)	TAACGTGGCCAAAATGATGC	GTTCTCCACAACCGCTTGGT	61 bp	(Czechowski *et al.*, 2005)
At4g26410 (RT-qPCR)	GAGCTGAAGTGGCTTCCATGAC	GGTCCGACATACCCATGATCC	81 bp	(Czechowski *et al.*, 2005)
UBQ10 (RT-qPCR)	AGAACTCTTGCTGACTACAATATCCAG	ATAGTTTTCCCAGTCAACGTCTTAAC	107 bp	This work

Table S2 Transcript abundances of the Rubisco gene family in *rbcs* mutants and transgenic lines of *Arabidopsis thaliana*. Abundances of *rbcS1A*, *rbcS1B*, *rbcS2B*, *rbcS3B* and *rbcL* transcripts were quantified using RT-qPCR with gene-specific primers (Table S1). Values are the means ± SE of measurements made on three 28-d-old rosettes (as shown in Fig. 2). For each subunit, letters above the means ± SE indicate significant difference (P <0.05) as determined by ANOVA followed by Tukey's HSD tests. Values followed by the same letter are not statistically significantly different.

	wild-type	*1a3b*	*1a2b*	1A$_{At}$-1	1A$_{At}$-2	1A$_{At}$-3	1A$_{At}$MOD-1	1A$_{At}$MOD-2	1A$_{At}$MOD-3	S2$_{Cr}$-1	S2$_{Cr}$-2	S2$_{Cr}$-3
rbcS1A	42.8 ± 1.2^{a}	0.08 ± 0.03^{b}	0.05 ± 0.02^{b}	0.02 ± 0.01^{b}	0.02 ± 0.01^{b}	0.02 ± 0.01^{b}	0.02 ± 0.01^{b}	0.02 ± 0.01^{b}	0.04 ± 0.03^{b}	0.04 ± 0.02^{b}	0.02 ± 0.01^{b}	0.02 ± 0.01^{b}
rbcS1B	8.1 ± 0.2^{a}	5.7 ± 1.6^{a}	9.8 ± 1.7^{a}	6.4 ± 2.5^{a}	10.3 ± 2.3^{a}	9.8 ± 2.2^{a}	8.2 ± 1.4^{a}	6.9 ± 1.8^{a}	4.6 ± 3.5^{a}	4.7 ± 2.8^{a}	7.7 ± 1.6^{a}	9.7 ± 2.4^{a}
rbcS2B	20.9 ± 2.7^{b}	34.0 ± 1.3^{ab}	0.01 ± 0.01^{c}	30.8 ± 8.8^{ab}	22.3 ± 5.2^{ab}	36.3 ± 9.8^{ab}	43.3 ± 8.4^{a}	21.4 ± 5.3^{b}	19.1 ± 3.4^{b}	33.7 ± 3.9^{ab}	42.1 ± 4.3^{a}	34.7 ± 6.3^{ab}
rbcS3B	28.2 ± 3.9^{a}	2.1 ± 0.8^{b}	31.7 ± 10.6^{a}	0.3 ± 0.1^{b}	0.1 ± 0.1^{b}	3.1 ± 0.9^{b}	0.5 ± 0.4^{b}	0.4 ± 0.2^{b}	0.5 ± 0.2^{b}	0.5 ± 0.4^{b}	0.2 ± 0.1^{b}	0.1 ± 0.1^{b}
rbcL	100 ± 6.1^{a}	48 ± 6.1^{c}	45 ± 2.1^{c}	86.2 ± 7.7^{ab}	94.5 ± 4.7^{ab}	109 ± 12^{a}	95.0 ± 3.6^{ab}	82.8 ± 9.2^{b}	92.1 ± 4.4^{ab}	98.3 ± 2.1^{a}	94.3 ± 5.2^{ab}	91.5 ± 2.1^{ab}
1A$_{At}$		-	-	45.1 ± 1.8^{a}	45.7 ± 4.2^{a}	43.3 ± 1.4^{a}	-	-	-	-	-	-
1A$_{At}$MOD		-	-	-	-	-	42.3 ± 2.1^{a}	46.7 ± 0.7^{a}	47.1 ± 3.3^{a}	-	-	-
S2$_{Cr}$		-	-	-	-	-	-	-	-	39.2 ± 1.7^{a}	42.2 ± 1.5^{a}	42.5 ± 0.8^{a}

New Phytologist

Table S3 Rubisco and soluble protein contents for *rbcs* mutants and transgenic lines of *Arabidopsis thaliana*. Rubisco content was determined via ^{14}C-CABP binding, subunit ratios were estimated by immunoblotting. Values are the means ± SE of measurements made on leaf samples from three 32-d-old rosettes (as shown in Fig. 3) followed by letters indicating significant difference (P <0.05) as determined by ANOVA followed by Tukey's HSD tests. Values followed by the same letter are not statistically significantly different.

	WT	*1a3b*	*1a2b*	1A$_{At}$-1	1A$_{At}$-2	1A$_{At}$-3	1A$_{At}$MOD-1	1A$_{At}$MOD-2	1A$_{At}$MOD-3	S2$_{Cr}$-1	S2$_{Cr}$-2	S2$_{Cr}$-3
Total Rubisco (g m^{-2})	0.86 ± 0.05^{a}	0.26 ± 0.08^{e}	0.42 ± 0.06^{de}	0.86 ± 0.7^{a}	0.58 ± 0.06^{bcd}	0.48 ± 0.1^{bcd}	0.53 ± 0.04^{bcd}	0.61 ± 0.1^{abcd}	0.76 ± 0.08^{ab}	0.56 ± 0.07^{bcd}	0.47 ± 0.07^{cd}	0.66 ± 0.06^{abc}
Soluble protein (g m^{-2})	2.9 ± 0.2^{a}	1.6 ± 0.2^{d}	1.9 ± 0.1^{cd}	2.6 ± 0.1^{ab}	2.5 ± 0.2^{ab}	2.4 ± 0.1^{abc}	2.4 ± 0.1^{abc}	2.6 ± 0.2^{ab}	2.8 ± 0.1^{ab}	2.5 ± 0.1^{ab}	2.3 ± 0.1^{abc}	2.6 ± 0.1^{ab}
LSU (g m^{-2})	0.68 ± 0.3^{a}	0.21 ± 0.05^{e}	0.34 ± 0.04^{de}	0.67 ± 0.05^{a}	0.45 ± 0.04^{bcd}	0.38 ± 0.07^{bcd}	0.42 ± 0.03^{bcd}	0.48 ± 0.08^{abcd}	0.59 ± 0.06^{ab}	0.44 ± 0.06^{bcd}	0.37 ± 0.05^{cd}	0.52 ± 0.05^{abc}
Total SSU (g m^{-2})	0.2 ± 0.01^{a}	0.05 ± 0.01^{e}	0.09 ± 0.01^{de}	0.18 ± 0.02^{ab}	0.13 ± 0.01^{bcd}	0.11 ± 0.03^{bcd}	0.12 ± 0.01^{bcd}	0.15 ± 0.03^{abc}	0.18 ± 0.02^{ab}	0.12 ± 0.02^{cd}	0.1 ± 0.01^{cd}	0.15 ± 0.01^{abc}
LSU: SSU (g: g)	3.4 ± 0.2^{a}	3.9 ± 0.2^{a}	3.8 ± 0.2^{a}	3.8 ± 0.3^{a}	3.4 ± 0.2^{a}	3.3 ± 0.2^{a}	3.6 ± 0.2^{a}	3.2 ± 0.3^{a}	3.2 ± 0.2^{a}	3.9 ± 0.3^{a}	3.7 ± 0.2^{a}	3.5 ± 0.3^{a}
Native SSU (g m^{-2})	0.2 ± 0.01^{a}	0.05 ± 0.01^{e}	0.09 ± 0.01^{cd}	0.18 ± 0.02^{ab}	0.13 ± 0.01^{bc}	0.11 ± 0.03^{cd}	0.04 ± 0.01^{e}	0.05 ± 0.01^{e}	0.04 ± 0.01^{e}	0.07 ± 0.01^{de}	0.06 ± 0.01^{e}	0.08 ± 0.01^{de}
Heterologous SSU (g m^{-2})	-	-	-	-	-	-	0.07 ± 0.01^{bc}	0.10 ± 0.03^{ab}	0.14 ± 0.02^{a}	0.05 ± 0.01^{c}	0.04 ± 0.01^{c}	0.06 ± 0.01^{bc}

Table S4 Rosette area and biomass for *rbcs* mutants and transgenic lines of *Arabidopsis thaliana*. Values are the means ± SE of measurements made on ten 28-d-old rosettes (as shown in Fig. 4). Letters above the means ± SE indicate significant difference (*P* <0.05) as determined by ANOVA followed by Tukey's HSD tests. Values followed by the same letter are not statistically significantly different. Abbreviations: FW, fresh weight; DW, dry weight; SLA, specific leaf area.

	WT	*1a3b*	*1a2b*	$1A_{At}$-1	$1A_{At}$-2	$1A_{At}$-3	$1A_{At}$MOD-1	$1A_{At}$MOD-2	$1A_{At}$MOD-3	$S2_{Cr}$-1	$S2_{Cr}$-2	$S2_{Cr}$-3	$1A_{At}$-1 seg	$1A_{At}$MOD-1 seg	$S2_{Cr}$-1 seg
FW (g)	0.27 ± 0.01^{a}	0.025 ± 0.003^{fg}	0.06 ± 0.01^{ef}	0.24 ±0.01^{ab}	0.24 ± 0.01^{ab}	0.2 ± 0.01^{bcd}	0.19 ± 0.01^{cd}	0.19 ± 0.01^{cd}	0.22 ± 0.01^{bc}	0.1 ± 0.01^{e}	0.16 ± 0.01^{d}	0.17 ± 0.01^{d}	0.02 ± 0.01^{g}	0.01 ± 0.01^{g}	0.01 ± 0.01^{g}
DW (mg)	24.5 ± 1.0^{a}	2.0 ± 0.1^{g}	6.1 ± 0.4^{f}	22.0 ±1.2^{ab}	21.4 ± 1.2^{abc}	18.5 ± 1.1^{bcde}	17.6 ± 1.0^{cde}	17.0 ± 1.0^{de}	20.3 ± 0.7^{bcd}	9.3 ± 0.4^{ef}	15.5 ± 0.9^{e}	16.3 ± 0.8^{e}	1.1 ± 0.1^{g}	1.0 ± 0.1^{g}	0.7 ± 0.1^{g}
FW/DW	11.2 ± 0.4^{ab}	12.8 ± 0.8^{a}	9.6 ± 0.1^{b}	10.8 ±0.3^{ab}	11.0 ± 0.3^{ab}	10.6 ± 0.1^{ab}	10.7 ± 0.1^{ab}	10.9 ± 0.1^{ab}	10.7 ± 0.1^{ab}	11.1 ± 0.6^{ab}	10.3 ± 0.1^{ab}	10.5 ± 0.1^{ab}	11.9 ± 1.3^{ab}	13.0 ± 1.0^{a}	12.1 ± 1.0^{ab}
Area (mm^2)	491 ± 14^{a}	89 ± 6^{d}	284 ± 21^{c}	487 ±23^{a}	455 ± 23^{ab}	433 ± 28^{ab}	445 ± 21^{ab}	411 ± 24^{ab}	458 ± 13^{ab}	273 ± 15^{c}	383 ± 19^{b}	437 ± 25^{ab}	50 ± 6^{d}	60 ± 3^{d}	47 ± 4^{d}
Area/FW (m^2 kg^{-1})	1.8 ± 0.1^{c}	3.7 ± 0.22^{b}	4.9 ± 0.4^{a}	2.1 ±0.1^{c}	2.0 ± 0.1^{c}	2.2 ± 0.1^{c}	2.3 ± 0.1^{c}	2.2 ± 0.1^{c}	2.1 ± 0.1^{c}	2.7 ± 0.2^{bc}	2.4 ± 0.1^{c}	2.6 ± 0.1^{bc}	5.1 ± 0.3^{a}	5.0 ± 0.3^{a}	4.8 ± 0.3^{a}
SLA (m^2 kg^{-1}) Area/DW	20 ± 0.1^{c}	47 ± 0.2^{b}	47 ± 0.4^{b}	22 ±0.1^{c}	21 ± 0.1^{c}	23 ± 0.1^{c}	25 ± 0.1^{c}	24 ± 0.1^{c}	23 ± 0.1^{c}	29 ± 0.1^{c}	25 ± 0.1^{c}	27 ± 0.1^{c}	48 ± 0.5^{ab}	63 ± 0.3^{a}	58 ± 0.4^{ab}

Table S5 Chlorophyll contents and maximum quantum yield of PSII (F_v/F_m) for *rbcs* mutants and transgenic lines of *Arabidopsis thaliana*. Values are the means ± SE of measurements made on four 28-d-old rosettes for chlorophyll and ten 28-d old rosettes for F_v/F_m rosettes. F_v/F_m is shown for attached leaves dark-adapted for 45 min prior to fluorescence measurements. Letters above the means ± SE indicate significant difference (P <0.05) as determined by ANOVA followed by Tukey's HSD tests. Values followed by the same letter are not statistically significantly different.

	WT	*1a3b*	*1a2b*	$1A_{At}$-1	$1A_{At}$-2	$1A_{At}$-3	$1A_{At}$MOD-1	$1A_{At}$MOD-2	$1A_{At}$MOD-3	$S2_{Cr}$-1	$S2_{Cr}$-2	$S2_{Cr}$-3
Chl a (µmol m^{-2})	185 ± 19[a]	82±4[b]	205 ± 3[a]	179 ± 3[a]	158 ± 12[a]	197 ± 13[a]	209 ± 12[a]	176 ± 13[a]	181 ± 5[a]	174 ± 10[a]	202 ± 13[a]	173 ± 9[a]
Chl b (µmol m^{-2})	59 ± 5[a]	26±2[b]	62 ± 2[a]	59 ± 1[a]	52 ± 4[a]	63 ± 2[a]	62 ± 3[a]	60 ± 2[a]	61 ± 2[a]	52 ± 3[a]	63 ± 2[a]	57 ± 1[a]
Chl a+b (µmol m^{-2})	245 ± 24[a]	108±5[b]	267 ± 3[a]	239 ± 3[a]	211 ± 16[a]	260 ± 15[a]	271 ± 15[a]	235 ± 15[a]	242 ± 4[a]	225 ± 13[a]	265 ± 14[a]	231 ± 9[a]
Chl a/b ratio	3.1 ± 0.1[a]	3.2±0.1[a]	3.3 ± 0.1[a]	3.0 ± 0.1[a]	3.0 ± 0.1[a]	3.1 ± 0.1[a]	3.4 ± 0.1[a]	2.9 ± 0.1[a]	3.0 ± 0.2[a]	3.4 ± 0.1[a]	3.2 ± 0.1[a]	3.0 ± 0.1[a]
F_v/F_m	0.854 ± 0.001[a]	0.764 ± 0.008[b]	0.856 ± 0.01[a]	0.850 ± 0.001[a]	0.85 ± 0.002[a]	0.85 ± 0.001[a]	0.846 ± 0.002[a]	0.841 ± 0.002[a]	0.852 ± 0.001[a]	0.846 ± 0.001[a]	0.848 ± 0.001[a]	0.849 ± 0.001[a]

Table S6 Photosynthetic nonphotochemical quenching capacity for *rbcs* mutants of *Arabidopsis thaliana*. Total NPQ was measured after 45 min exposure to high light (600 µmol photons m^{-2} s^{-1}) and after 1 h darkness. Rapidly relaxing NPQ (NPQ_{slow}) and slowly relaxing NPQ (NPQ_{fast}) were quantified according to Griffiths & Maxwell (1999). Values are the means ± SE of measurements on individual leaves from three different rosettes, followed by letters indicating significant difference (*P* <0.05), as determined by ANOVA followed by Tukey's HSD tests. Values followed by the same letter are not statistically significantly different.

	WT	*1a3b*	*1a2b*
NPQ capacity	3.39 ± 0.32^{b}	4.33 ± 0.28^{a}	2.19 ± 0.21^{c}
% NPQ_{fast} (qE)	1.84 ± 0.11^{b} (55%)	3.39 ± 0.4^{a} (78%)	0.72 ± 0.25^{c} (32%)
% NPQ_{slow} (qI)	1.55 ± 0.32^{a} (45%)	0.94 ± 0.28^{c} (22%)	1.47 ± 0.1^{b} (68%)

References

Czechowski T, Stitt M, Altmann T, Udvardi MK, Scheible WR. 2005. Genome-wide identification and testing of superior reference genes for transcript normalization in Arabidopsis. *Plant Physiology* **139**: 5–17.

Griffiths H, Maxwell K. 1999. In memory of C. S. Pittendrigh: Does exposure in forest canopies relate to photoprotective strategies in epiphytic bromeliads? *Functional Ecology* **13**: 15–23.

Izumi M, Tsunoda H, Suzuki Y, Makino A, Ishida H. 2012. RBCS1A and RBCS3B, two major members within the Arabidopsis RBCS multigene family, function to yield sufficient Rubisco content for leaf photosynthetic capacity. *Journal of Experimental Botany* **63**: 2159–2170.

Karimi M, Inze D, Depicker A. 2002. GATEWAY vectors for *Agrobacterium*-mediated plant transformation. *Trends in Plant Science* **7**: 193–195.

Nakagawa T, Ishiguro S, Kimura T. 2009. Gateway vectors for plant transformation. *Plant Biotechnology* **26**: 275–284.
