# Supplementary material for: Rubisco small subunits from the unicellular green alga Chlamydomonas complement Rubisco‐deficient mutants of Arabidopsis
Source: New Phytol. 2017 Jan 13;214(2):655–67. doi: 10.1111/nph.14414 (PMC5363358; doi:10.1111/nph.14414)
Supplement: Supplementary file 2 — Notes S1 Expression vectors for Rubisco small subunit (rbcS) cassettes. [file NPH-214-655-s002.zip › Notes S1.docx]

**Notes S1** Expression vectors for Rubisco small subunit (*rbcS*) cassettes (archived .zip file). Gateway destination vector pB7WG (Karimi *et al*., 2002) was used for stable Agrobacterium-mediated insertion into Arabidopsis. For fluorescent tag-based localisation in tobacco, *rbcS* genes were fused to a sequence encoding a GFP tag using destination vector pGWB4 (Nakagawa *et al*., 2009), to produce C-terminally GFP-tagged fusion protein.
